# The Pattern of Copper Release in Copper‐Based Nanoparticles Regulates Tumor Proliferation and Invasiveness in 3D Culture Models

**DOI:** 10.1002/smsc.202400206

**Published:** 2024-08-27

**Authors:** Jose I. Garcia‐Peiro, Paula Guerrero‐López, Felipe Hornos, Jose L. Hueso, J. Manuel Garcia‐Aznar, Jesus Santamaria

**Affiliations:** ^1^ Instituto de Nanociencia y Materiales de Aragon (INMA) CSIC‐Universidad de Zaragoza Campus Rio Ebro Edificio I+D C/ Poeta Mariano Esquillor, s/n 50018 Zaragoza Spain; ^2^ Department of Chemical and Environmental Engineering University of Zaragoza Campus Rio Ebro C/María de Luna, 3 50018 Zaragoza Spain; ^3^ Networking Research Center in Biomaterials, Bioengineering and Nanomedicine (CIBER‐BBN) Instituto de Salud Carlos III 28029 Madrid Spain; ^4^ Instituto de Investigación Sanitaria (IIS) de Aragón Avenida San Juan Bosco, 13 50009 Zaragoza Spain; ^5^ Multiscale in Mechanical and Biological Engineering (M2BE) Aragon Institute of Engineering Research (I3A) University of Zaragoza Mariano Esquillor s/n 50018 Zaragoza Spain; ^6^ Instituto de Investigación, Desarrollo e Innovación en Biotecnología Sanitaria de Elche (IDiBE) Universidad Miguel Hernández Av. de la Universidad s/n 03202 Elche Alicante Spain; ^7^ Escuela Politécnica Superior Universidad de Zaragoza Crta. de Cuarte s/n 22071 Huesca Spain

**Keywords:** 3D cultures, copper, core‐shell, glioblastoma, microchips, release

## Abstract

Cancer is a leading cause of death worldwide. Glioblastoma (GBM) is a major challenge in oncology due to its highly invasive nature and limited treatment options. GBM's aggressive migration beyond tumor margins and rapid tumor growth hinders success in patient treatment. Localized therapeutic delivery, such as the use of transition metals like copper, is highlighted as a novel therapeutic agent for many potential biomedical applications. Herein, it is aimed to study the effects of Cu release on the proliferation and invasiveness of cancer cells. To this end, novel copper‐based nanostructures with different release patterns are designed. Using a complex 3D cell culture model to mimic the tumor microenvironment, it is shown that different patterns of copper ion release have a strong impact on GBM progression and invasiveness. The findings highlight the importance of optimizing localized copper release patterns to tailor different tumor treatment strategies. They also show the potential and suitability of 3D microchips as instruments to study the behavior of tumor spheroids. In spite of their limitations, these 3D microdevices enable a controlled and close monitoring of the influence of environmental factors (such as the presence of Cu ions) on the proliferation and invasiveness of the cells, with a better approach to reality compared to 2D models and with a more controlled environment, compared to an in vivo model.

## Graphical abstract

Copper on 3D culture models: the influence of copper released from nanoparticles on glioblastoma can be explored in 3D cell scaffolds cultured in microfluidic chips. This work highlights the importance of optimizing localized copper release patterns to tailor different tumor treatment strategies. They also show the potential and suitability of 3D microchips as instruments to study the behavior of tumor spheroids.

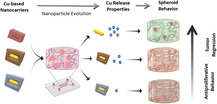

## 1 Introduction

Cancer is one of the leading causes of death in the world and the search for effective treatment remains a challenge in modern medicine.^[^ ^1^ ^]^ This disease induces uncontrolled cell growth, evasion of cell death mechanisms, and the ability to invade surrounding tissues.[^1^, ^2^] In particular, glioblastoma (GBM) is a great cancer model as it is one of the most malignant cancers^[^ ^3^ ^]^ and is considered a predominant brain cancer disease, where only 5% of patients survive after 5 years.^[^ ^4^ ^]^ The aggressiveness of this malignancy lies in its tendency to invade adjacent tissues.^[^ ^5^ ^]^ The local infiltration and migration result in an elusive disorder where conventional therapeutic approaches remain insufficient.^[^ ^6^ ^]^ GBM demonstrates a tumor cell migration pattern where both individual and collective cells infiltrate nearby tissues, out of the tumor margins, limiting surgery efficiency.^[^ ^7^ ^]^ Tumor progression is highly linked with the tumor microenvironment (TME) where extracellular matrix (ECM) plays a key role.^[^ ^8^ ^]^ Interestingly, GBM rarely metastasizes to other organs possibly due to the features of brain TME as blood‐brain barrier.^[^ ^9^ ^]^ Thus, a proper understanding of tumor progression with special emphasis on the matrix contribution is fundamental to develop novel therapeutic agents that control tumor growth and invasiveness for single and combined therapy.[^5^, ^8^, ^10^]

In the last few years, nanoparticles (NPs) have been in the spotlight due to their relevance as potential therapeutic candidates.[^1^, ^11^] Cisplatin and other related compounds have been applied in clinics as effective DNA chelators.^[^ ^12^ ^]^ Transition metals such as copper, have gained more attention due to their implications in copper‐dependent cell growth and proliferation (cuproplasia).^[^ ^13^ ^]^ Excess copper via copper supplementation can cause toxicity related to oxidative stress and reactive oxygen species (ROS) generation.^[^ ^14^ ^]^ However, the elevated systemic toxicity and unfavorable pharmacokinetics of copper have driven researchers to explore alternative Cu‐based carriers to circumvent these restrictions.^[^ ^15^ ^]^ Indeed, disulfiram (DSF) or elesclomol (ES), two copper ionophores are used as therapeutics to induce cuproptosis.^[^ ^13^, ^15^, ^16^ ^]^

The development of copper carriers has been explored beyond molecular chelators and other delivery alternatives including engineered nanodevices have been tested as Cu suppliers.[^15^, ^17^] Polymeric nanoplatforms with copper ion reservoirs have been widely explored as nanocarriers.^[^ ^18^ ^]^ They behave as good biodegradable and biocompatible devices with proper mechanical properties; however, their drug‐loading ability remains limited.^[^ ^19^ ^]^ Alternatively, copper‐based NPs including copper oxide, sulfide, or bimetallic NPs are able to increase the effectiveness of copper delivery.[^15^, ^20^] The major drawback appears in the lack of understanding about the factors governing the release of Cu ionic species from copper‐based nanomaterials.[^15^, ^17^, ^21^] In fact, the implementation and discussion of dissolution properties of ions in nanomedicine is far from being standardized and well‐defined.^[^ ^22^ ^]^ Studies of Cu‐based NPs as anticancer therapeutic agents usually test cytotoxicity in 2D cell culture models.^[^ ^23^ ^]^ However, this culture approach exhibits certain limitations such as the loss of cell–cell and cell–extracellular environment interactions or of cellular heterogenicity,^[^ ^24^ ^]^ which makes it an inefficient model for drug testing.^[^ ^25^ ^]^ In recent years, 3D models have been used to study nanotoxicology in cancer because of the remarkable advantages to monitor critical parameters such as cell organization or protein corona formation.^[^ ^26^ ^]^ Therefore, 3D cellular models represent a step forward in nanomedicine development where ECM accounts as a key parameter. Likewise, animal experimentation is reduced and increases the chances of success in preclinical trials.^[^ ^8^, ^25^, ^26^ ^–^ ^27^ ^]^

**Figure 1:**
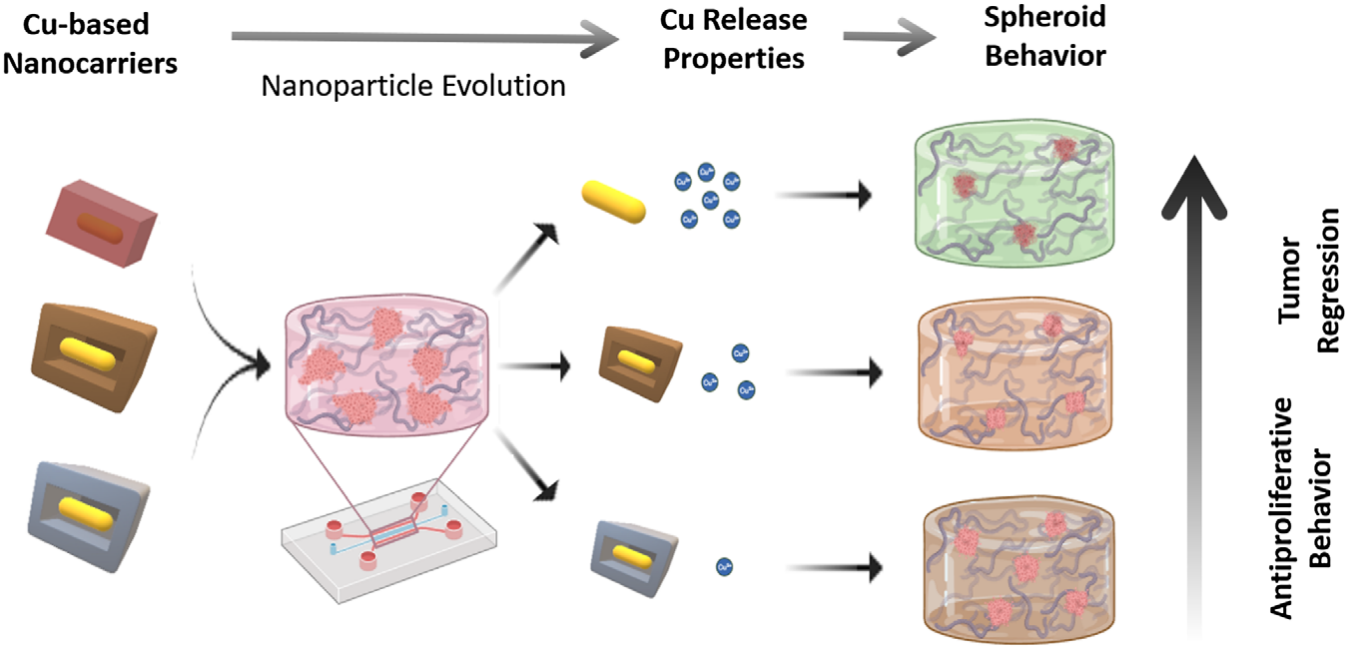
Schematic illustration of tumor evolution under the influence of different Cu‐release nanocarriers. Impact of copper release intensity on tumor progression and invasiveness.

Here, we study the effects of Cu release on the proliferation and invasiveness of cancer cells. To this end, we have designed novel copper‐based nanostructures with different release patterns, depending on the exposure to different biologically relevant media (see **Figure** **1**). We have evaluated the influence of each media and the relevance of the release behavior in tumor progression, including proliferation and organization. We studied the impact of copper released in a GBM 3D cell culture model in a microfluidic device that allowed us to quantitatively assess both the invasive and proliferative potentials of GBM cancer cells. The results of our study clearly relate the extent and intensity of Cu release with a strong decrease in the proliferation and invasiveness of 3D GBM spheroids opening up new therapeutic possibilities. The 3D‐used approach represents a clear advantage over other 2D/3D culture models in the literature,^[^ ^28^ ^]^ because it allows tumor cells to self‐organize together with an ECM‐like environment that regulates tumor progression. Notwithstanding the simplified nature of 3Ds compared to the real 3D TME, it provides a more representative platform to test the potential of new treatments on tumor progression, such as the Cu‐releasing NPs used in this work.

## 2 Results

### 2.1 Tuning Release Patterns of Copper‐Based Nanocarriers

**Figure 2:**
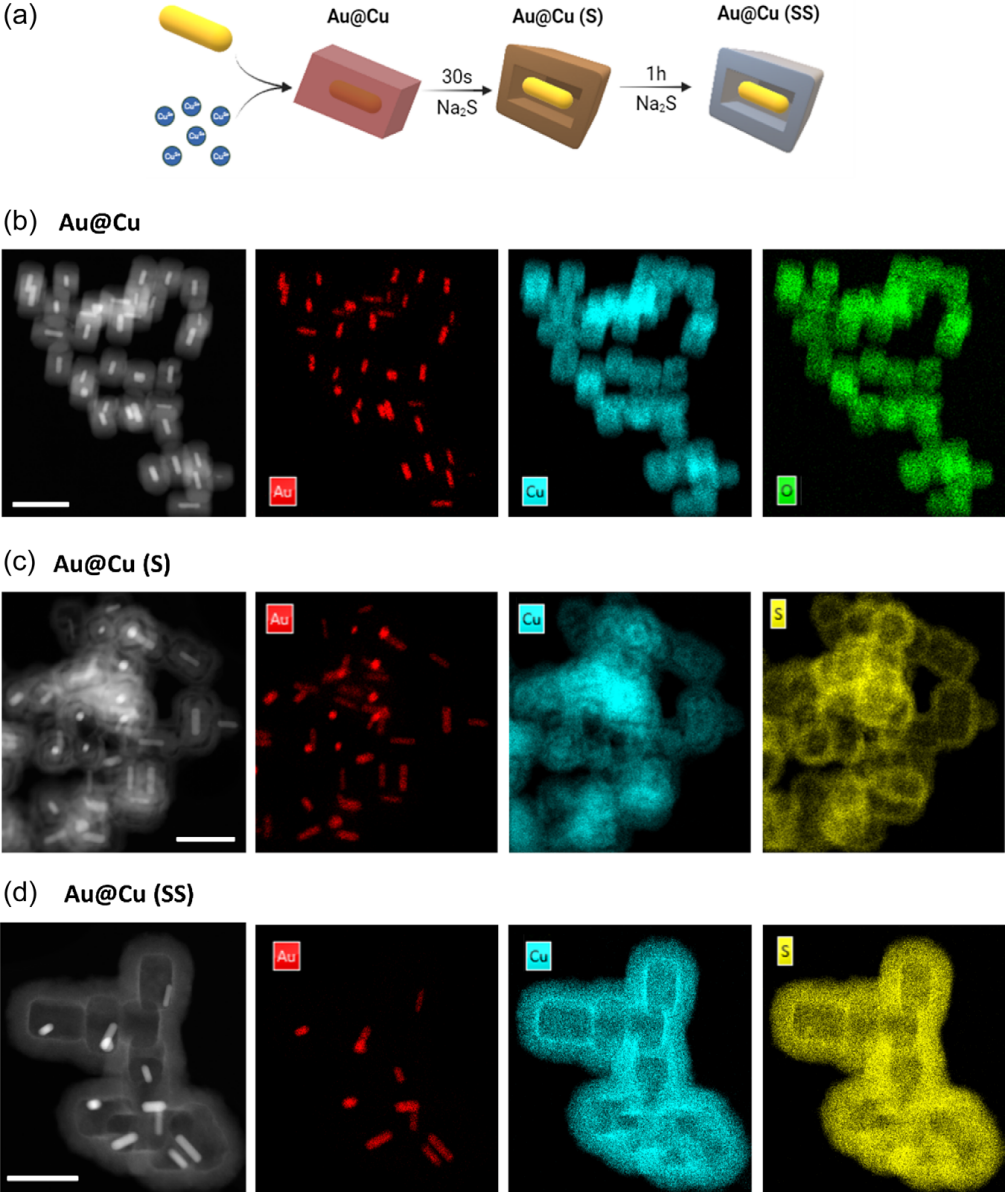
HAADF‐STEM–EDX analysis of the different Cu‐based nanostructures: a) schematic illustration of the synthetic protocol for different Cu‐based NPs where the Au nanorods at the core define crystal growth and Na_2_S promote sulfidation to form Au@Cu, Au@Cu (S) and Au@Cu (SS); HAADF‐STEM images (left) and corresponding elemental mapping energy‐dispersive spectroscopy (EDS) analysis of: b) Au@Cu NPs with core‐shell configuration (Au is located at the core and Cu and O are found around in the external shell); c) Au@Cu (S) NPs with core‐shell configuration where Au is placed in the innermost core, S and Cu are found together forming the external shell while a core of Cu_2_O remains unreacted around the Au nanorod; and d) Au@Cu (SS) NPs with a rattle‐like configuration, where the Au nanorod can be found in the empty inner space and Cu and S are colocalized in the shell. Scale bar = 200 nm.

We developed a robust synthesis protocol to help modulate copper release kinetics. Cu_2_O NPs and Cu‐based nanocarriers with a core‐shell configuration were synthesized to tune Cu release properties (see **Figure** 1–**2** and Figure S1–S2, Supporting Information). First, we optimized the Cu release rate by tuning NP size. In the structural configuration designed, we observed that Cu release is a surface‐driven phenomenon, and its speed increases as the particle becomes smaller and the surface‐to‐volume ratio increases. Second, the total amount of Cu released could also be adjusted by changing the elemental composition of Cu‐based nanocarriers. Cu oxides such as Cu_2_O NPs completely release Cu ions in the presence of physiological stimuli such as serum, overexpressed glutathione ([GSH] = 5 mM), or acidic pH values (6).^[^ ^17^, ^20^ ^]^ In contrast, Cu sulfides are less prone to lixiviation under similar biological conditions. A controlled sulfidation step could be therefore proposed to control and diminish the release rate of Cu and alleviate its intrinsic toxicity.

To finely control Cu release kinetics, we used Au nanorods (Au NRs) as core directing seeds. A clear correlation was found between the Au/Cu metallic ratio and the size of the core‐shell hybrid Au@Cu NPs. The larger the concentration of Au NR seeds, the smaller the diameter of the surrounding Cu‐based shell (see transmission electron microscopy (TEM) images in Figure 2 and Figure S1–S2, Supporting Information). Furthermore, on this core‐shell structure, we developed two levels of sulfidation by treating Au@Cu NPs with Na_2_S for 30 s and 1 h to yield new configurations denoted as Au@Cu (S) and Au@Cu (SS) NPs, respectively, to indicate different degree of sulfidation (Figure 2). High‐angle annular dark field combined with scanning transmission electron microscopy (HAADF‐STEM) analysis confirmed that the neat core‐shell configuration of Au@Cu NPs was progressively transformed into a rattle‐like structure as an amorphous CuS shell was progressively formed by transformation of Cu_2_O with increasing sulfidation time for Au@Cu (S) and Au@Cu (SS) (Figure 2c,d and Figure S3, Supporting Information). This enlarged the total size of the NP, and gradually loosened the Au nanorod within the rattle‐like configuration (Figure 2b–d).

Elemental analysis (energy‐dispersive X‐Ray spectroscopy (EDX)) of these three samples (Figure 2b–d) confirmed the presence of Au in the internal core for every sample. Au@Cu NPs exhibited a shell composition of Cu and O (Figure 2 and Figure S2, Supporting Information). Single NP analysis displayed a Cu/O ratio of 2 (Figure S2, Supporting Information) and no sulfur was detected in the sample. In contrast, EDX analysis of Au@Cu (S) NPs revealed the presence of Cu under two different scenarios: Cu in the external shell always colocalized with sulfur atoms while at the core only unreacted Cu with no sulfur in the structure was found (Figure 2c and Figure S3, Supporting Information). In addition, EDX mapping analysis of Au@Cu (SS) NPs revealed a total sulfidation of Cu to form a CuS shell around an internal void containing the nanorod, in a rattle‐like shape configuration (Figure 2d). The evolution of sulfidation can be described as a process where the reaction proceeds from the outside to the inside until the Cu_2_O core is transformed (Figure 2). UV–vis analysis of the different samples corroborated the change of the composition around the AuNRs and how the second absorption maxima at longer wavelengths progressively disappeared upon increasing sulfidation treatment (Figure S4b, Supporting Information). Likewise, X‐Ray diffraction (XRD) analysis also confirmed the presence of metallic Au with cubic structure and the progressive disappearance of the (111) and (200) diffraction peaks of the Cu_2_O cubic phase,^[^ ^29^ ^]^ thereby confirming the transformation of the oxide phase and the substitution by an amorphous CuS shell (Figure S4, Supporting Information).

**Figure 3:**
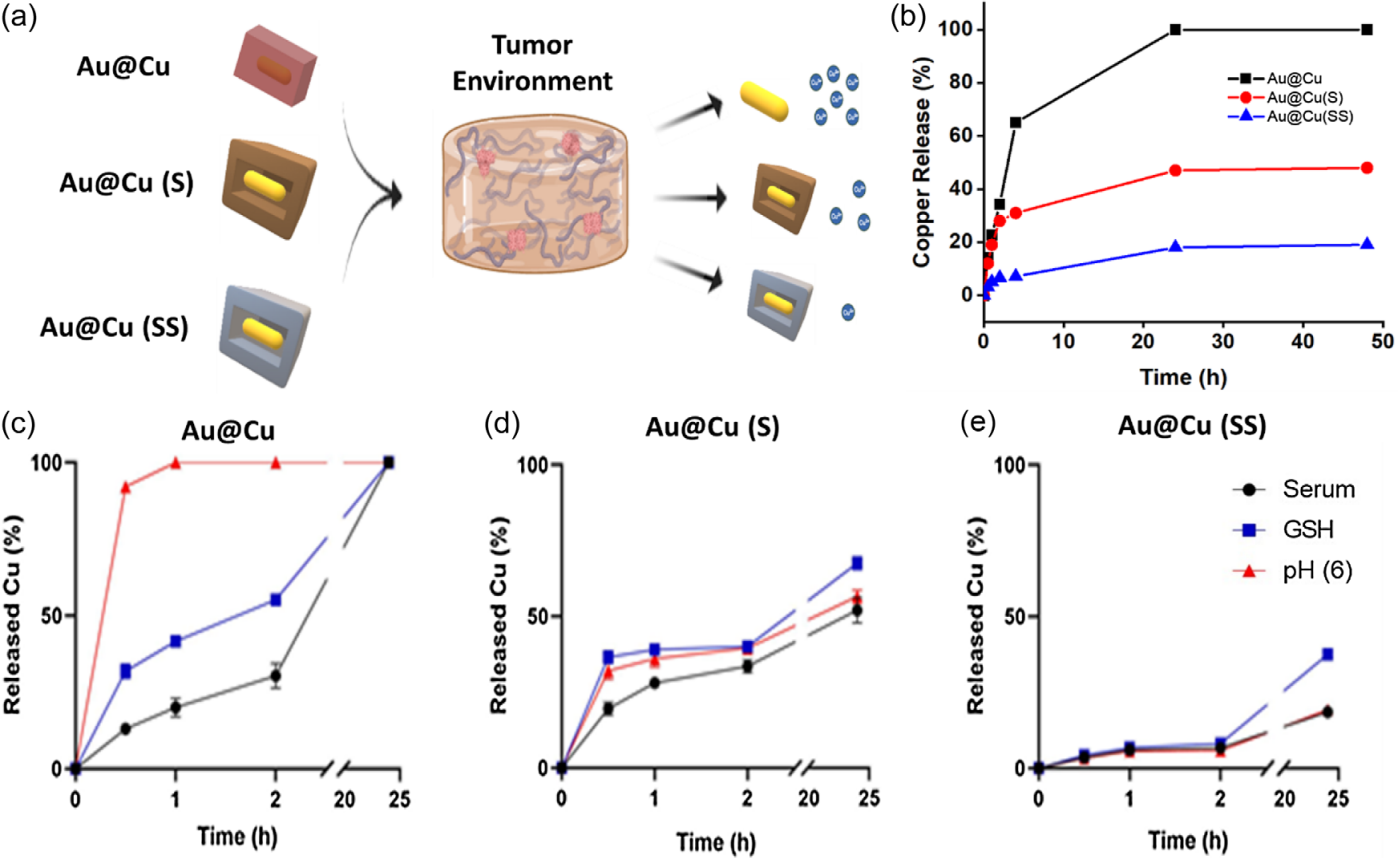
Copper release from the different Cu‐based nanostructures in the presence of different triggering stimuli: a) schematic illustration of copper release properties of Cu‐based NPs (Au@Cu, Au@Cu (S) and Au@Cu (SS)) under the influence of different parameters characteristic of the physiological environment. b) Effect of sulfidation on release rate: comparison of Au@Cu, Au@Cu (S), and Au@Cu (SS) NPs in serum over time. After 24 h exposure, Cu release was established in a plateau at levels of 100%, 52%, and 19% of the total Cu content, respectively, depending on the degree of sulfidation. c–e) Cu release from c) Au@Cu NPs, d) Au@Cu (S), and e) Au@Cu (SS) NPs when exposed to such as serum, acidic conditions (pH = 6), and GSH (5 mM).

Next, we evaluated the release properties of the different Cu‐based configurations under relevant physiological conditions (**Figure** **3**a). The influence of size in the release kinetics of Cu_2_O‐based NPs was studied with nonsulfured NPs, including Cu_2_O NPs with no Au core. As expected, the larger the Cu_2_O NPs, the slower the release rates when exposed to fetal bovine serum (FBS) (Figure S5, Supporting Information). It can be seen that after 48 h exposure to serum, every Cu_2_O‐based NPs reached 100% of Cu release. This is in sharp contrast with the behavior of Na_2_S‐treated NPs (Au@Cu (S) and Au@Cu (SS) NPs). When exposed to FBS each of them reached a maximum release that decreased with sulfidation: the 100% release observed with Au@Cu decreased to 55% and 19% for Au@Cu (S) and Au@Cu (SS) NPs, respectively (Figure 3b). In addition to the maximum amount released, we studied the kinetics release of the Au@Cu, Au@Cu (S) and Au@Cu (SS) core‐shell nanostructures under relevant physiological conditions. Specifically, the release kinetics observed in serum were compared to those under acidic (pH = 6) and in the presence of GSH, at concentrations representative in the TME ([GSH] = 5 mM).^[17b]^

Au@Cu NPs revealed faster kinetics releases in the following scenarios pH 6 > GSH > serum (Figure 3c). In contrast, Au@Cu (S) NPs exhibited similar release properties for pH and serum but somewhat higher release rates in the presence of GSH, reaching 69% (compared to 52%) of Cu released cumulatively after 24 h (Figure 3d). The relative enhancement of Cu release in the presence of GSH was the highest for Au@Cu (SS) NPs when Cu release reached 38%, i.e., twice that obtained under serum and low pH (Figure 3e). Additional characterization by UV–vis spectroscopy, XRD analysis (Figure S6, Supporting Information) and advanced microscopy (Figure S7, Supporting Information) also confirmed the progressive transformation of the different Cu‐structures, the rattle‐like evolution of the core‐shell solid structures and the loss of the crystalline Cu_2_O phases. Therefore, the tunable selection of different degrees of sulfidation helped us to trigger different release profiles depending on the external stimuli conditions typically present in the TME. However, it must be noted that these experiments were performed in the collagen‐free medium. Since the experiments with spheroids were to be carried out in a microfluidic device filled with a collagen matrix that served as a support for spheroid growth, we also explored the effect of collagen on the Cu ions released. Therefore, we also repeated the Cu‐release experiments with the three types of NPs embedded in a collagen matrix. The results are displayed in Figure S8, Supporting Information. As it can be observed, most of the Cu was associated with the collagen, with only a smaller fraction (15%–20%) in the serum phase, regardless of the NP used and the total concentration of Cu released. This seems to imply a sort of equilibrium between Cu in serum and Cu adsorbed in the collagen matrix. Nevertheless, as shown later, the fraction of Cu in serum was sufficient to induce strong effects on the proliferation of tumor spheroids. In any case, the results highlighted the prevalence of Cu ions in the collagen phase, indicating that collagen is likely to affect metal ion dynamics and should be taken into account in future studies.

### 2.2 The Copper Release Pattern Regulates Tumor Spheroid Progression

The three types of Cu‐based NPs with different release capabilities were tested in a 3D cell culture model to assess the effect of Cu in tumor spheroid development as a simplified biological model of tumor progression. We used a microfluidic device^[^ ^30^ ^]^ made of polydimethylsiloxane (PDMS), with a central chamber containing a hydrogel based on collagen type I that acts as a matrix for cell culture and two side channels through which nutrients are introduced (Figure S9, Supporting Information). Initially, individual U251‐MG GBM cells were seeded with the corresponding Cu nanocarrier, embedded in the collagen‐based hydrogel, so tumor cells can proliferate and self‐organize three‐dimensionally according to the matrix architecture.^[^ ^30^ ^]^

**Figure 4:**
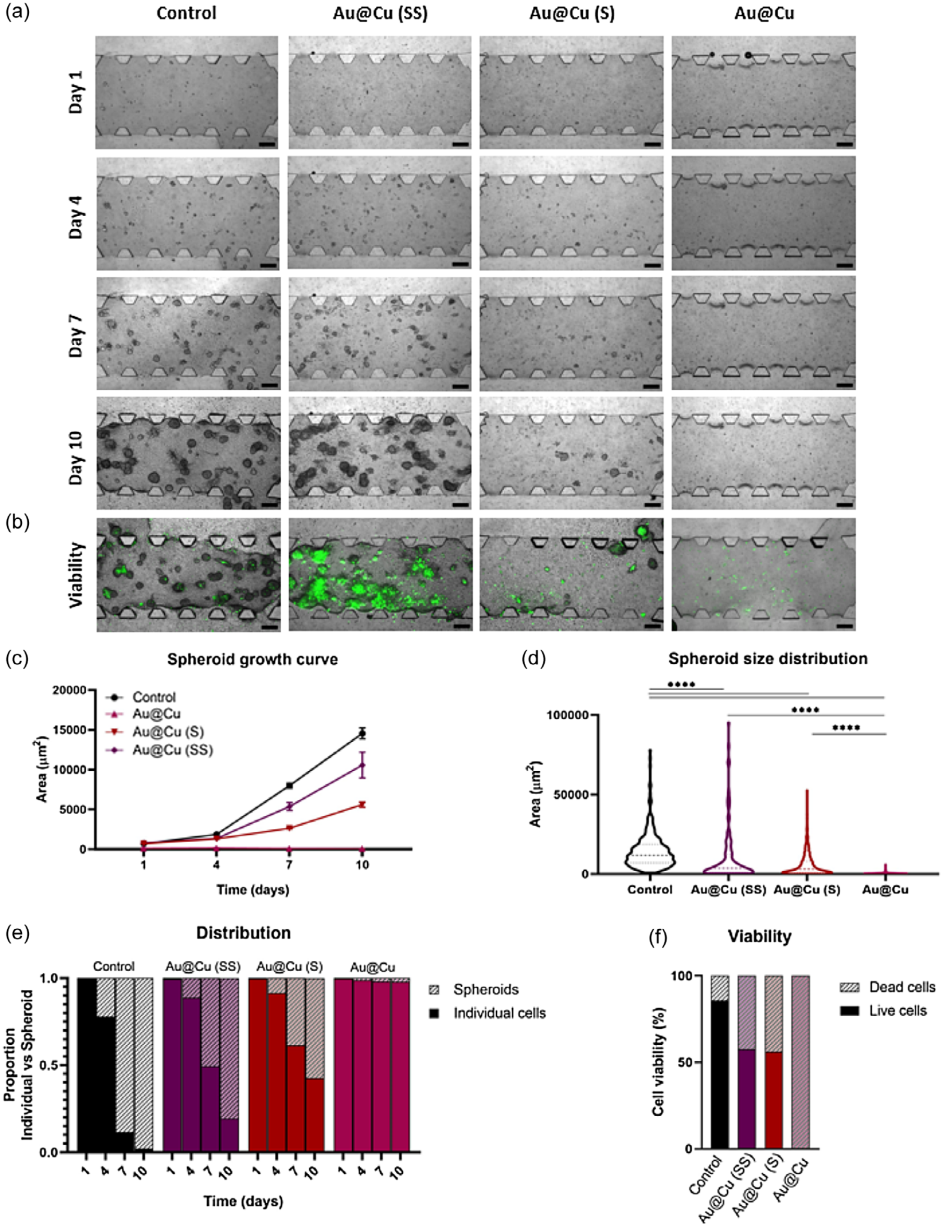
Influence of copper release pattern in 3D tumor growth: a) brightfield microscopy along 10 d (*n* = 3) allows the comparison of the growth of the 3D cellular cultures when exposed to a total concentration of 0.1 mg mL^−1^ using Cu‐based NPs with different release patterns. Scale bar is 250 μm. b) Cellular viability tested using SYTOX Green Nucleic Acid Stain. Scale bar is 250 μm. All fluorescence images were acquired with 488 nm (green) laser and have identical exposure times and normalization. c) Images were processed and segmented to obtain the growth curve, d) population size distribution at endpoint, and e) proportion of spheroid formation. f) Images obtained with the viability probe SYTOX Green Nucleic Acid Stain were quantified through segmentation to obtain the live/dead cell ratio. Data are shown as the mean ±  standard error of the mean (SEM) (*n* = 3, technical and experimental replicates are three each); *p‐val < 0.033; **p‐val < 0.002; ***p‐val < 0.001.

First, we investigated the cytotoxicity of Au@Cu and Au@Cu (SS) NPs at different concentrations in tumor and healthy cells, to assess any effects associated with specific cell types (Figure S10, Supporting Information). Our results indicate that the NPs exhibited lower cytotoxicity in human astrocytes than in the GBM cell line. Then, we studied the influence of Cu‐based NPs, on the formation and growth of tumor spheroids and compared them with those treated (**Figure** **4**a). The growth of tumor spheroids was greatly altered depending on the Cu leaching capacity of the NPs. This was reflected in the number and size of spheroids obtained in the different cultures. The population treated with Au@Cu (SS) NPs had a similar evolution as the control. However, tumor cells treated with Au@Cu NPs did not form any spheroid under the same total copper concentration. Spheroids treated with Au@Cu (S) NPs showed an intermediate behavior, with considerable depletion in size and number compared with nontreated populations (control) and populations treated with Au@Cu (SS) NPs.

To evaluate tumor progression, we monitored the total 2D‐projected area of tumor spheroids every 3 d (Figure 4c). A rapid tumor growth was observed for the nontreated group, while Au@Cu (SS) led to a slightly slower growth of tumors. In contrast, spheroids treated with Au@Cu and Au@Cu (S) displayed a strongly inhibited growth compared with other groups. This effect could be directly related to the copper release capacity. This 3D culture model allowed us to compare the size distribution of the tumor spheroids at the time‐point of interest. Figure 4d shows these distributions near the end of the study period (after 10 d), and again, very significant differences were obtained for the different treatments. Control devices had a wide variety of spheroid sizes, revealing significant differences even with respect to Au@Cu (SS) treated spheroids despite their low Cu leaching capacity, although a few very large spheroids could be observed in both cases. These results were in contrast with cultures treated with Au@Cu (S) NPs where a strong reduction in their size was observed. Devices treated with Au@Cu NPs displayed the strongest inhibition in total growth and size of individual spheroids (Figure 4c,d). The tendency of cells to assemble and form spheroids followed a parallel trend with the intensity of the Cu‐release treatment. Initially, all cells grew individually, but they rapidly formed spheroids for control and Au@Cu (SS) treatment (around 95% and 80% were organized as spheroids, respectively, at day 10), in strong contrast with Au@Cu(S) and Au@Cu, with 60% and almost no spheroid formation, respectively (Figure 4).

Finally, a live/dead analysis was performed using SYTOXGreen Nucleic Acid Stain fluorescence imaging to assess the toxicity induced by Cu release (Figure 4b and Figure S11, Supporting Information). The green fluorescence signal indicated that viability was regulated with copper released. Notably, while cells treated with Au@Cu (SS) had displayed similar trends regarding growth and spheroid formation to the control thanks to the low level of Cu lixiviation, there were significant differences in viability (Figure 4f), with 85% in the control versus 57% in the population treated with Au@Cu (SS), indicating that the presence of Cu ions affected cell viability even under relatively slow copper release conditions. It is also interesting to note that for samples treated with Au@Cu (S) and Au@Cu (SS), while they displayed the same viability, the spheroid growth and size distribution curves presented strong differences. This observation suggests that NPs mechanism not only affects viability but also modulates cancer cell proliferation and their ability to form spheroids.

### 2.3 The Copper Release Pattern Strongly Influences 3D Tumor Invasiveness

**Figure 5:**
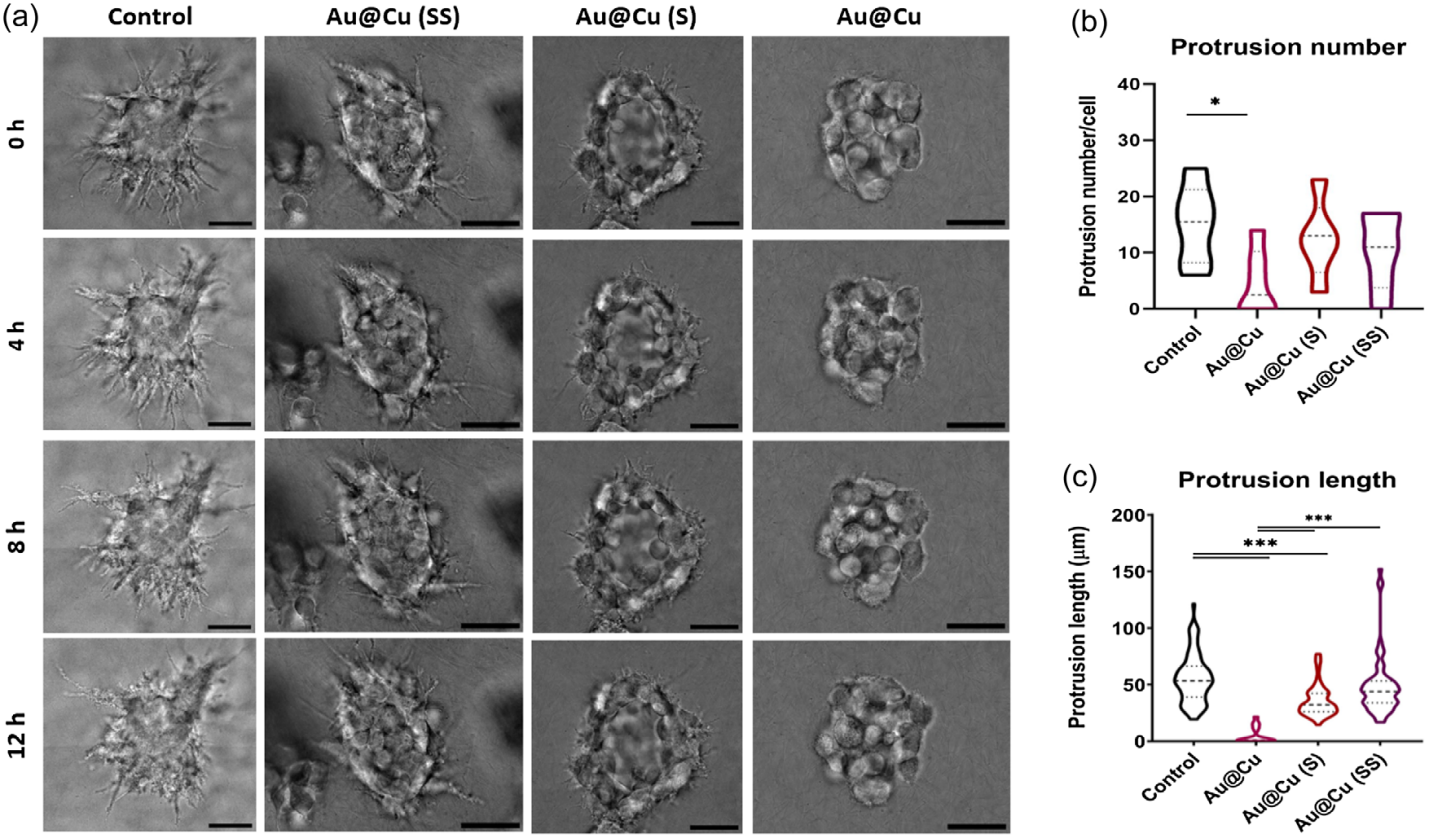
Influence of copper release pattern in protrusion formation in 3D tumors: a) brightfield images obtained from time lapses recorded for 12 h, after 9 d of incubation with NPs with a total copper concentration of 0.1 mg mL^−1^. Digital images acquired every 4 h. Scale bar is 50 μm. b) ImageJ analyzed protrusions of six different spheroids per treatment to obtain statistical analysis of protrusion number and c) length of six different spheroids per treatment. Data are shown as their distribution with median and the interquartile range (IQR) (*n* = 6 spheroids per condition); *p‐val < 0.033; **p‐val < 0.002; ***p‐val < 0.001.

GBM‐derived cells produce many protrusions in correspondence with their highly aggressive and invasive capacity, so we measured protrusion formation as a marker of tumor invasiveness^[^ ^31^ ^]^ after treatment with Cu‐based to evaluate its influence. To this aim, after 9 d of treatment inside the microfluidic device, we performed 12 h time‐lapses. During this period, spheroids with different phenotypes were formed due to the NPs treatment. This assay allowed us to evaluate protrusion formation, movement, and evolution of many spheroids after being subjected to the presence of Cu ions (treatment) or not (control) (**Figure** **5**a). This assay showed a strong decrease in the protrusion‐forming capacity of the spheroids, depending on the amount of Cu‐released in the TME. To perform a quantitative analysis, we evaluated different parameters such as protrusion number (Figure 5b) and maximum length (Figure 5c) per cancer spheroid. When branches were analyzed, there was only a slight decrease in the protrusion number if we compared control spheroids with the ones treated with sulfidized NPs. Only spheroids treated with Au@Cu NPs showed a significantly lower number of protrusions compared with the control sample or even with Au@Cu (S) and Au@Cu (SS) ones. Interestingly, there was a more acute effect when we focused on the maximum length of branches produced, where a clear correlation could be observed with the intensity of Cu release (Control> Au@Cu(SS)> Au@Cu(S)> Au@Cu).

**Figure 6:**
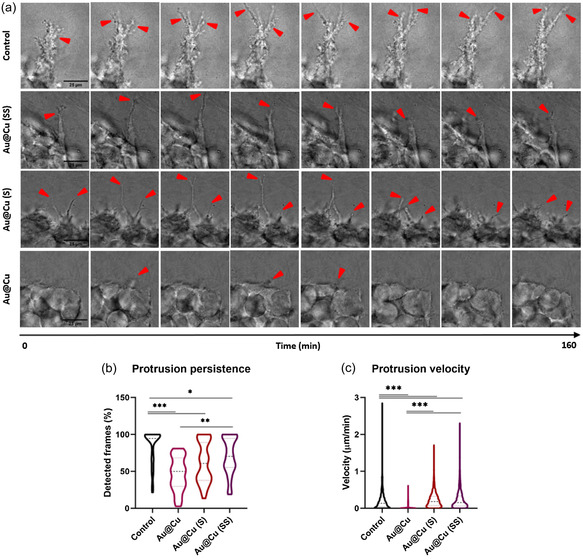
Influence of copper release pattern in protrusion formation and development in 3D tumors: a) brightfield images of the evolution of protrusions examples in each condition. The time between each image is 20 min, with a total interval of 2 h and 40 min. The images are an extract from time lapses recorded during 12 h, after 9 d of treatment with NPs with a total copper concentration of 0.1 mg mL^−1^. Red arrows point to example protrusions and their evolution depending on treatment. Scale bars = 25 μm. b) ImageJ analyzed protrusions of six different spheroids per treatment to obtain statistical analysis of persistence through time and c) velocity of protrusion movement. Data are shown as their distribution with median and the IQR (*n* = 6 spheroids per condition); *p‐val < 0.033; **p‐val < 0.002; ***p‐val < 0.001.

To gain insight into specific characteristics of protrusion formation under each condition, we additionally measured persistence and velocity. **Figure** **6**a illustrates an example of protrusion development for each treatment. Notably, control protrusions exhibited continuous growth, even branching at times. In contrast, treated cells displayed branches with reduced development directly correlated to the quantity of Cu released by the NPs. This effect was prominently established by the persistence parameter (Figure 6b) with control protrusions persisting throughout the study period while treated ones were not consistently detected. Protrusion velocity showed a similar correlation (Figure 6c), wherein spheroids treated with lower amounts of Cu exhibited reduced protrusion movement capability. These branches are constantly exploring the cluster's surroundings, leading to a continuous movement of reorganization and progression of the spheroid (Figure S12, Supporting Information). Therefore, increasing the amount of copper in the microenvironment damages spheroids protrusions and reduces tumor reorganization and progression movements (Movies S1–S4, Supporting Information), leading even to cell collapse in the case of the most extreme treatment (Movie S5, Supporting Information). To further study this effect, migration of individual cells (Figure S13 and Movies S6–S9, Supporting Information) as well as the invasiveness of the spheroids (Figure S14, Supporting Information) under the different treatments were studied. These assays highlighted the impact of NPs on the dissemination of individual cells from the original cell cluster, as both invasiveness and cell migration were strongly inhibited as a function of copper‐releasing capacity. These results indicate that the presence of copper in the microenvironment not only causes cell death but also decreases the invasiveness of spheroid by making the tumor grow in a less favorable environment.

### 2.4 The Copper Release Pattern Reduces 3D‐Spheroid Malignancy

**Figure 7:**
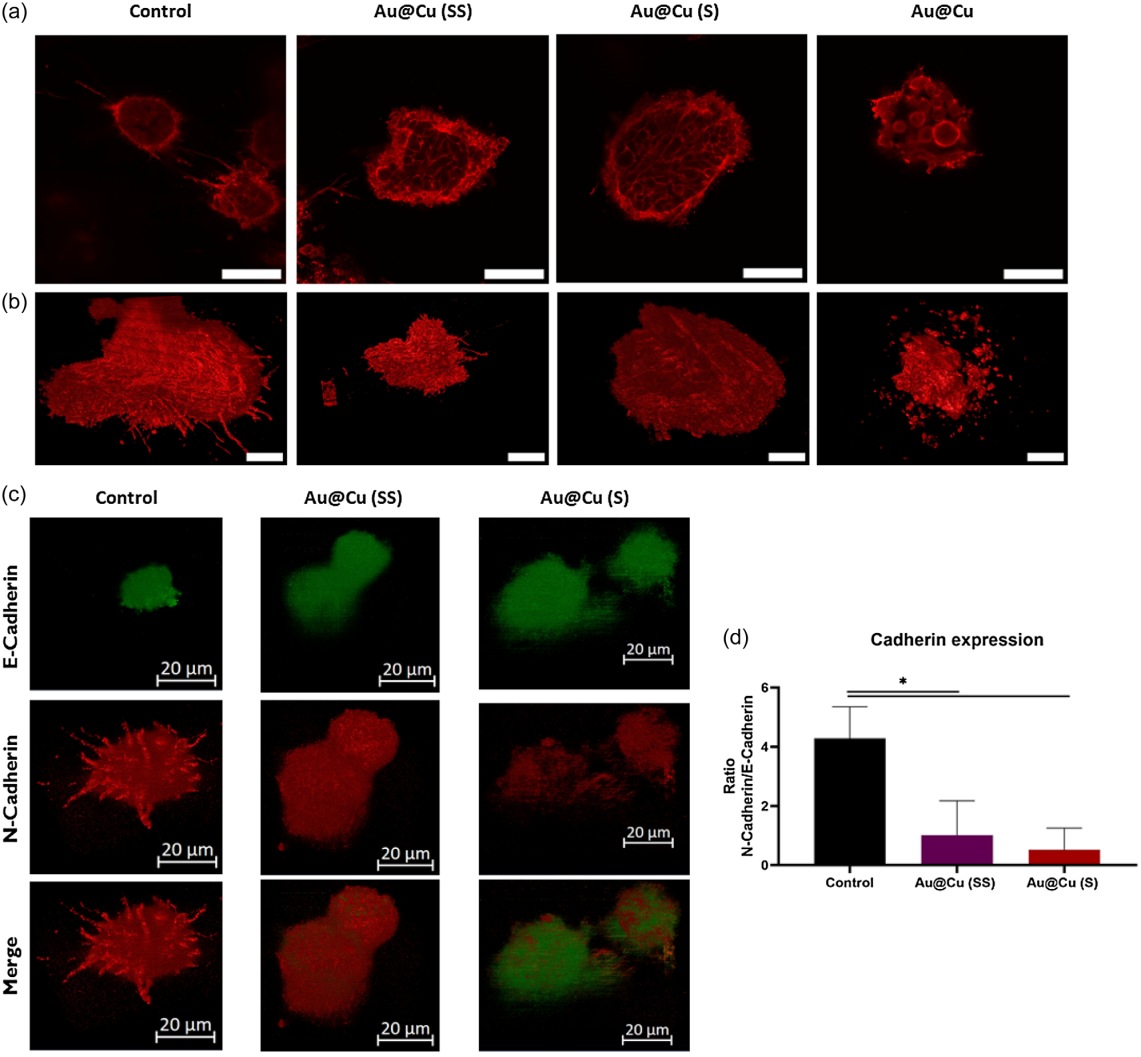
Malignancy reduction of 3D‐spheroids treated with Cu‐based NPs with different copper release patterns: a) 2D confocal images of phalloidin‐stained GBM spheroids treated with different Cu‐based NPs (scale bar = 50 μm) and b) 3D tumor reconstruction of phalloidin‐stained GBM spheroids treated with different Cu‐based NPs (scale bar = 20 μm). All fluorescence images were acquired with 561 nm laser and have identical exposure times and normalization. c) 3‐dimensional reconstruction of E‐ (green) and N‐Cadherin (red) expression and distribution over the spheroid (scale bar = 20 μm). All fluorescence images were acquired with 488 nm (green) and 640 nm (red) laser and have identical exposure times and normalization. d) Fluorescence intensity of the orthogonal projection was analyzed with MATLAB to obtain the ratio of expression under the different treatments. Data are shown as the mean ± SEM (*n* = 3); *p‐val < 0.033; **p‐val < 0.002; ***p‐val < 0.001.

To further determine the effect of Cu‐based nanocarriers over GBM malignancy, we evaluated different stains. First, we analyzed the nuclei and cytoskeleton of spheroids organization using confocal imaging acquisition (**Figure** **7**a) and 3D reconstruction techniques (Figure 7b), which allowed to perform a morphological study. Very relevant differences in spheroid architecture were revealed following treatment with different NPs. The nuclei analysis revealed no differences between treatments, except for those cells treated with Au@Cu NP, which exhibited notable alterations in nucleus morphology, suggesting substantial cellular damage (Figure S15, Supporting Information). Of note, the actine capsule in the control cells was strongly altered when treated with Cu‐based NPs, being even lost when spheroids were exposed to the most intense Cu release from Au@Cu NPs. In agreement with the results displayed in the previous section, we also noticed how the protrusions emitted by cells in the 3D reconstructions were notoriously altered when they were treated with Cu‐based NPs (Figure 7 and Movies S10–S13, Supporting Information). Tumor cells treated with Au@Cu NPs showed no full spheroid formation, only cell aggregates with a high mortality rate as can be seen by the high content of apoptotic bodies and cell debris. However, for intermediate‐intensity treatments such as Au@Cu (SS) and Au@Cu (S) NPs, the influence of copper over the spheroid architecture and protrusion formation could be clearly observed (Movies S10–S13, Supporting Information). Clearly, as the intensity of Cu release increased, the intensity of cell protrusion formation decreased accordingly and cell cytoskeleton appeared highly injured.

In addition, we evaluated the epithelial‐to‐mesenchymal transition (EMT) state to further study the aggressiveness of tumor spheroid.^[^ ^32^ ^]^ Thus, we tested the E‐ and N‐Cadherin expression of GBM‐derived cells spheroids as main markers of EMT (Figure 7c). In this case, cells treated with Au@Cu NPs were no*t* tested due to the elevated mortality. Spheroids grown in the absence of Cu‐based NPs showed a small inner E‐Cadherin (green) core surrounded by a highly predominant N‐Cadherin (red) coating. This distribution correlates with a highly aggressive spheroid.^[^ ^33^ ^]^ However, spheroids grown in the presence of Cu‐based NPs showed a predominant E‐Cadherin (green) expression when Au@Cu (S) NPs were used, even on the surface of the formation. Tumors treated with Au@Cu (SS) NPs also showed significant differences compared with control experiments (Figure 7d). This means that the Cu leached from the NPs makes the phenotype becomes more epithelia and the spheroid obtained is less aggressive than the untreated samples.

## 3 Discussion

The enhanced migratory potential of cancer cells is strictly correlated with aggressiveness, invasiveness, and, lately, poor prognosis in several cancer types as GBM.[^5^, ^34^] Solid tumors exhibit a complex and invasive behavior characterized by the uncontrolled proliferation of abnormal cells that infiltrate surrounding tissues. This invasion involves multiple steps, including the degradation of ECM components, enhanced cell motility, and the establishment of new blood vessels through angiogenesis.^[1c]^ In this way, TME plays a key role in tumor behavior as it provides a great niche for tumor growth, progression, and development.[^2^, ^30^] Patient‐specific tumor‐on‐chip models of human tumors, which attempt to simulate the pathological and complex characteristics of TME have been demonstrated very useful for the identification of effective treatments for GBM patients.^[8b]^ In this work, we have also presented a microfluidic‐based 3D cell culture that may offer advantages over other 1D, 2D, or 3D in vitro tests.[^8^, ^10^, ^31^] Traditionally, other 3D approaches are based on preformed multicellular aggregates in a matrix‐free plate, which are embedded in a 3D matrix.^[^ ^35^ ^]^ However, in our engineered microfluidic approach, we seed individual cells into a 3D matrix to promote their proliferation and the corresponding self‐organization of tumor cells guided by the microarchitecture of the collagen‐based hydrogel. Thus, tumor cells are able to construct their own TME through proliferation and their interaction with the collagen‐based matrix, shaping their own tumor niche.^[^ ^30^, ^36^, ^37^ ^]^ Nevertheless, we want to point out that these spheroids, 3D cultures in microchips are still a simplified model far from fully representing the complex reality of tumor growth. Likewise, we are convinced that the use of spheroids grown in a collagen matrix is significantly better than a 2D model.

Herein, we have evaluated and quantified the growth and the dynamics of invasion of GBM‐derived spheroids when treated with different Cu‐based NPs and different metal release patterns. Our work offers a rational approach to modulate the intensity of the release of copper ions using different copper‐based NPs. To this end, we engineered a protocol where Cu_2_O‐based NPs can release 100% of their Cu content as ions. However, a greater degree of release control can be achieved with an additional sulfidation step of the Cu_2_O shells upon reaction with Na_2_S. We could also modify the release kinetics of copper by turning NPs size using a seed‐mediated protocol. This novel synthetic approach effectively tunes both the total Cu released and the kinetic rate of ionic Cu leaching. We have further tested three different NPs with 100%, ≈50%, and ≈20% Cu release features in our cancer‐on‐chips devices. We have found that the intensity of Cu release directly regulates the progression of GBM (U251‐MG) spheroids, as well as their invasiveness and malignancy capacity. The temporal size and shape of tumor spheroids and the formation and size of protrusions were quantified. The formation of these protrusions seems to orchestrate the tumor cell dissemination during metastasis.^[^ ^38^ ^]^ Here, we demonstrate that the peaked release and persistence of Cu ions direct spheroids toward a less aggressive phenotype, significantly reducing the number and size of invasive protrusions.

## 4 Conclusion

Our insights will help us to better understand the role of ECM in cargo release under physiological conditions and will better replicate in vivo cellular progression with on‐time monitoring of cancer spheroid behavior. Although our 3D model hinders the study of molecular markers, it allows us to study other relevant biophysical markers that go unnoticed with other techniques. In this way, we have evaluated spheroids growth and malignancy with protrusions formation, highlighting this parameter as an important invasiveness and metastatic marker. Indeed, our model's obstacles can be tackled in the future, and engineered models present a promising option to accurately define cargo release dynamic and their further influence in dissecting the mechanisms of tumor progression with special emphasis on the evaluation of the biomechanical and biophysiological properties of solid tumors. Still, the present study provides new insights and a new perspective regarding the mechanochemical behavior in GSM cells grown in a 3D environment and the influence of released copper beyond its direct impact on GSH depletion and ROS generation already discussed in the recent literature.

## 5 Experimental Section

5.1

5.1.1

##### Materials

Hydrogen tetrachloroaurate (AuHCl_4_·3H_2_O, 50% basis), copper (II) chloride dihydrate (CuCl_2_·2H_2_O, ≥ 99.0%), sodium acetate anhydrous (CH_3_COONa), bovine serum albumin (BSA), phosphate‐buffered saline (PBS) buffer (pH 7,4), glutathione (≥98, HPLC), hydrogen peroxide (H_2_O_2_, 33% v/v), hexadecyltrimethylammonium bromide (CTABr) (96%), sodium borohydride (99,99%), L‐ascorbic acid (99%), 5‐bromosalicylic acid (90%), silver nitrate (99.9999%), hydroxylamine hydrochloride (HONH_2_·HCl), sodium hydroxides (NaOH), sodium dodecylsulfate (SDS), polyvinylpyrrolidone (PVP, M.W. = 40,000 Da), poly‐D‐lysine (PDL), and phalloidine were purchased from Sigma Aldrich. Water was obtained from a Milli‐Q Advantage A10 System with resistivity of 18.2 mΩ (Merk Millipore, Germany). Polydimethylsiloxane (PDMS, Sylargd 184) was obtained from Dow Corning GmbH, Dulbecco's modified Eagle's medium (DMEM) and FBS from Gibco, and collagen type I (rat tail high concentration) from Corning. Antibodies for N‐Cadherin (Mouse mAb) and E‐Cadherin (Rabbit mAb) were purchased from Cell Signaling Technology, and their respective secondary antibodies Alexa Fluor 647 (Goat antimouse IgG) and Alexa Fluor 488 (Goat anti‐Rabbit IgG), as well as SYTOX Green Nucleic Acid Stain from Life Technologies (ThermoFisher). Invitrogen supplied Dapi.

##### Synthesis of Copper‐Based NPs: Preparation of the Au@Cu Nanostructures

To prepare Au@Cu NPs, we synthesized a Cu_2_O‐based NP with copper release ability. Typically, CuCl_2_·2H_2_O (0.5 mL, 0.1 M) was first added into water (100 mL). Second, sodium dodecyl sulfate (1 g) was added under stirring. Then, NaOH (1.25 mL, 1 M) and NH_2_OH·HCl (3.5 mL, 0.1 M) were immediately added to the mixture. The resulting solution was then shaken gently for 20 s and subsequently left undisturbed for 1 h at room temperature. To modulate the kinetic properties of Cu_2_O‐based NPs and control the NPs growth, we used Au‐based cores. Au cores used as growing seeds were prepared according to previously reported protocols without any further modifications.^[^ ^39^ ^]^ Size‐controlled Cu‐based NPs were synthesized analogously and washed two times with water by centrifugation to remove the surfactant. Preparation of the Au@Cu (S)/Au@Cu (SS) nanostructures: Au@Cu NPs were dispersed in 10 mL of MilliQ water. Then, 100 mg of PVP was added to the mixture and we stirred for 2 min. Next, 5 mg of Na_2_S was added to the reaction. The resulting solution was gently stirred for 30 s or 1 h to obtain Au@Cu (S)/Au@Cu (SS), respectively. The NPs were centrifuged and washed twice with DI water for purification.

##### Characterization Techniques

TEM was performed using a FEI TECNAI T20 microscope operated at 200 keV. Aberration‐corrected STEM (Cs‐corrected STEM) images were acquired using a high‐angle annular dark field detector in a FEI XFEG TITAN electron microscope (Hillsboro, Oregon) operated at 300 kV and equipped with a CETCOR Cs‐probe corrector from CEOS GmbH (Heidelberg, Germany), allowing the formation of an electron probe of 0.08 nm. The geometric aberrations of the probe‐forming system were controlled to allow a beam convergence of 24.7 mrad half‐angle. Elemental analysis was carried out with an EDX detector for energy‐dispersive spectroscopy experiments in scanning mode. EDX mappings were acquired with an Oxford Instruments (NanoAnalysis & Asylum Research, High Wycombe, UK) detector and analyzed with the built‐in AZtec software. The sample was prepared by drop casting 5 μL of the NP suspension on a Ni holey carbon TEM grid. X‐Ray photoelectron spectroscopy (XPS) was performed with an Axis Supra spectrometer (Kratos Tech). The samples were mounted on a sample rod placed in the pretreatment chamber of the spectrometer and then evacuated at room temperature. The spectra were excited by a monochromatized Al Kα source at 1486.6 eV and subsequently run at 8 kV and 15 mA. A survey spectrum was measured at 160 eV of pass energy, and for the individual peak regions, spectra were recorded with a pass energy of 20 eV. Analysis of the peaks was performed with the CasaXPS software using a weighted sum of Lorentzian and Gaussian component curves after Shirley background subtraction. The binding energies were referenced to the internal C 1*s* standard at 284.5 eV. XRD patterns were obtained in a PANalytical Empyrean equipment in Bragg‐Brentano configuration using CuKα radiation and equipped with a PIXcel1D detector.

##### Copper Release Kinetics

Each solution was prepared in a 3 mL well (24 multiwell plates) with Cu NPs at a concentration of 0.1 mg mL^−1^ (total volume = 2 mL). The different solutions were stirred at 400 rpm. At every time point, a 200 μL sample was centrifuged at 10 000 rpm for 2 min. Pellets and supernatants were collected in different Eppendorfs for further analysis. The NP pellet was resuspended with the corresponding amount of water. At the experiment endpoint, the supernatant samples were analyzed together, to close mass balances and elucidate how much metal moved to the solution. All the samples were digested with HCl:HNO_3_ (3:1) mixture overnight. Cu concentrations were determined through the analysis with Agilent 4100 MP‐AES.

##### Microfluidic Device Fabrication

The microfluidic devices used are made of PDMS with a design consisting of a central chamber where one hydrogel, which recreates tissue matrix, is confined and two side channels through which the nutrients are introduced, as shown in Figure S9, Supporting Information. The geometry was adjusted in an SU‐8 master mold on a silicon wafer, from which it was replicated with PDMS. This material is fabricated with a 10:1 weight ratio mixture of base and curing agent, cured in an oven at 80 °C, and then cropped and punched to make the access to the channels. Finally, the PDMS devices were attached to the glass bottom of 35 mm Petri dishes by activating the surfaces with a plasma treatment and, later, they were treated with PDL to improve the adhesion of the collagen matrix to the device.

##### Hydrogel Preparation and Cell Seeding

The human GBM cell line U251‐MG was cultured with DMEM at 4.5 g L^−1^ glucose and supplemented with 10% FBS. Cells were incubated at 37 °C with 5% CO_2_ until 80% confluence was reached for use in the experiment. For seeding in the three‐dimensional culture, they were trypsinized, centrifuged (1200 rpm, 5 min), and passed through a 40 μm cell strainer to ensure the removal of cell aggregates. Subsequently, cells were counted using a Neubauer chamber and added to the collagen mix to leave a final concentration of 0.2 × 10^6^ cell mL^−1^. The three‐dimensional cell culture was developed in a type I collagen‐based matrix using the protocol by Shin et al.^[^ ^40^ ^]^ Following these indications, the hydrogel consisted of a mixture at 4 °C of 10X DPBS, collagen at a final concentration of 6 mg mL^−1^, 0.5 M NaOH to adjust pH to 7.5, the cells and the NPs at a copper concentration of 0.1 mg mL^−1^, previously sonicated and homogenized. This mix was introduced into the central chamber, as shown in Figure S9a, Supporting Information and left to polymerize at 37 °C in humid boxes, turning the device every 5 min for at least 20 min. Finally, the lateral channels were hydrated with a culture medium periodically.

##### Image Acquisition and Analysis

The spheroid growth was monitored with a Leica DM IL Led microscope. Photos were taken of the central chamber daily at 4X magnification in brightfield. Later, these images were processed and the spheroids area was segmented with the semiautomatic Segmentation3D App developed by C. Borau using MATLAB (Mathworks, Natick, CA, US) as described by Alamán‐Díez et al.^[^ ^41^ ^]^ The data obtained were processed and represented using GraphPad Prism 8. Fluorescence images of viability were taken also with the Leica DM IL Led microscope. For the spheroid migration, 12‐h time lapses after 9 d of treatment were performed with a Carl Zeiss Axio Observer Z1 7. Photos were taken every 20 min at 40X magnification in brightfield at 37 °C with 5% CO_2_, and the migration analysis was done with Fiji‐ImageJ and MATLAB, as described by Plou et al.^[^ ^30^ ^]^ Protrusions were analyzed using the same time lapses acquired. Measurements of protrusion length were performed with ImageJ. 2D structure fluorescence images were obtained using the Nikon D‐Eclipse C1 confocal microscope equipped with a Plan Apo VC 40XH objective and for 3D reconstruction of the cytoskeleton ZEISS Lattice Lightsheet 7 microscope was used at 40X magnification.

##### Immunofluorescence Staining and Viability Staining

For the structure analysis, the samples were stained with DAPI and Phalloidine. To begin with, samples were fixed with 4% paraformaldehyde in PBS for 15 min and to remove it, 5 min washes were performed 3 times. To permeabilize cells membrane, samples were treated for 10 min at room temperature with 0.1% Triton X‐100 in PBS and, after that, washed 3 times with PBS for 5 min. Blocking was done with 5% BSA in PBS overnight at 4 °C. Later, phalloidine and DAPI were added to samples, both of them diluted 1:100 in PBS, and incubated for 4 h at room temperature in darkness. Cells were washed 3 times for 5 min with PBS again. For the EMT study, E‐ and N‐Cadherin were stained in spheroids, using Alexa 488 and 647, respectively. The staining protocol is similar to structure one, with primary and secondary antibodies. Fluorescence intensity was measured with MATLAB. Cellular viability was tested using SYTOX Green Nucleic Acid Stain, a probe that penetrates cells with compromised plasma membranes and bounds to nucleic acid. The final concentration in the DMEM culture medium was 0.1 μM. Cells were incubated overnight at 37 °C inside the microdevices. Fluorescence images were taken with the Leica DM IL Led microscope.

##### Cytotoxicity Assay

Human astrocytes and the human GBM cell line U251‐MG were cultured with human astrocyte medium (Innoprot) and DMEM, respectively, at 4.5 g L^−1^ glucose and supplemented with 10% FBS. Cells were incubated at 37 °C with 5% CO_2_ until 80% confluence was reached for use in the experiment. Cells were seeded in the microdevice as above with Au@Cu and Au@Cu (SS) NPs in the initial hydrogel mixture. Three concentrations of each NP were used: 0.2, 0.1, and 0.025 mg mL^−1^. Cells were incubated for 7 d and cytotoxicity was measured with CellTiter‐Glo Luminescent Cell Viability Assay (Promega). CellTiter‐Glo reagent was added to the microdevices in a mixture 1:1 with DMEM. After 30 min of incubation at RT, the medium was collected in a dark 96‐well plate and luminescence was measured using a plate reader

##### Individual Cell Migration Assay

To study individual cell migration, U251‐MG cells were seeded as described above inside our microfluidic devices in 2.5 mg mL^−1^ collagen type I hydrogel containing 0.1 mg mL^−1^ of the respective NP. After seeding, the cells were incubated for 24 h at 37 °C to allow acclimatization to the system. Then, time‐lapse imaging was performed by acquiring brightfield images every 20 min for 24 h. The incubation conditions were controlled at 37 °C, 5% CO_2_, and 95% humidity. Approximately 50 cells in each set of experimental samples were tracked. Cell trajectory acquisition was performed using the Manual Tracking tool from ImageJ. Data processing with a MATLAB script developed by Moreno‐Arotzena et al.^[^ ^42^ ^]^ allowed us to extract the cell mean (*V*_mean_) and effective (*V*_eff_) velocities. Note that Vmean is defined as the averaged instantaneous speed including all time steps, whereas *V*_eff_ takes into account only the initial and final positions.

##### Invasiveness Assay

U‐251 MG cell spheroids were cultured by plating 1000 viable cells per well in 100 μL using an ultra‐low attachment 96‐well plate for 72 h. The formed spheroids were then collected and each was embedded in 10 μL of 2.5 mg mL^−1^ collagen type I hydrogel containing 0.1 mg mL^−1^ of the respective NP. The hydrogel drops containing the spheroid were placed in a 24‐well plate and supplied with 100 μL of DMEM. Migration was observed at 24, 48, 72, and 96 h using brightfield microscopy, and images were processed with ImageJ to measure the spheroid and its respective invasion area.

##### Statistical Analysis

Each condition underwent triplicate testing. Statistical analysis was conducted using GraphPad Prism 8 and expressed as the mean ± SEM. The normality of the data was assessed using the Shapiro‐Wilk test. Analysis of variance (ANOVA) was then performed, followed by post hoc Dunnett tests to ascertain statistical significance across the continuous variables under different conditions. Two‐way ANOVA followed by Dunnett multiple comparison test was used to analyze data with more than one variable. In cases where data distribution was non‐normal, nonparametric Kruskal–Wallis tests were employed, followed by post hoc Dunn's tests. All statistical tests performed are two‐tailed, and a *p*‐value of < 0.05 is considered significant.

## Conflict of Interest

The authors declare no conflict of interest.

## Supporting information

Supplementary Material

## Data Availability

The data that support the findings of this study are available in the supplementary material of this article.
