# Supplementary material for: The Pattern of Copper Release in Copper‐Based Nanoparticles Regulates Tumor Proliferation and Invasiveness in 3D Culture Models
Source: Small Sci. 2024 Aug 27;4(12):2400206. doi: 10.1002/smsc.202400206 (PMC11935094; doi:10.1002/smsc.202400206)
Supplement: Supplementary file 1 — Supplementary Material [file SMSC-4-2400206-s001.zip › smsc.202400206-sup-0001-suppdata-S1.pdf]

# Supporting Information

**The pattern of copper release in copper-based nanoparticles regulates tumor proliferation and invasiveness in 3D culture models**

*Jose I. Garcia-Peiro⁑, Paula Guerrero-López⁑, Felipe Hornos, Jose L. Hueso*, J. Manuel Garcia-Aznar* and Jesus Santamaria**

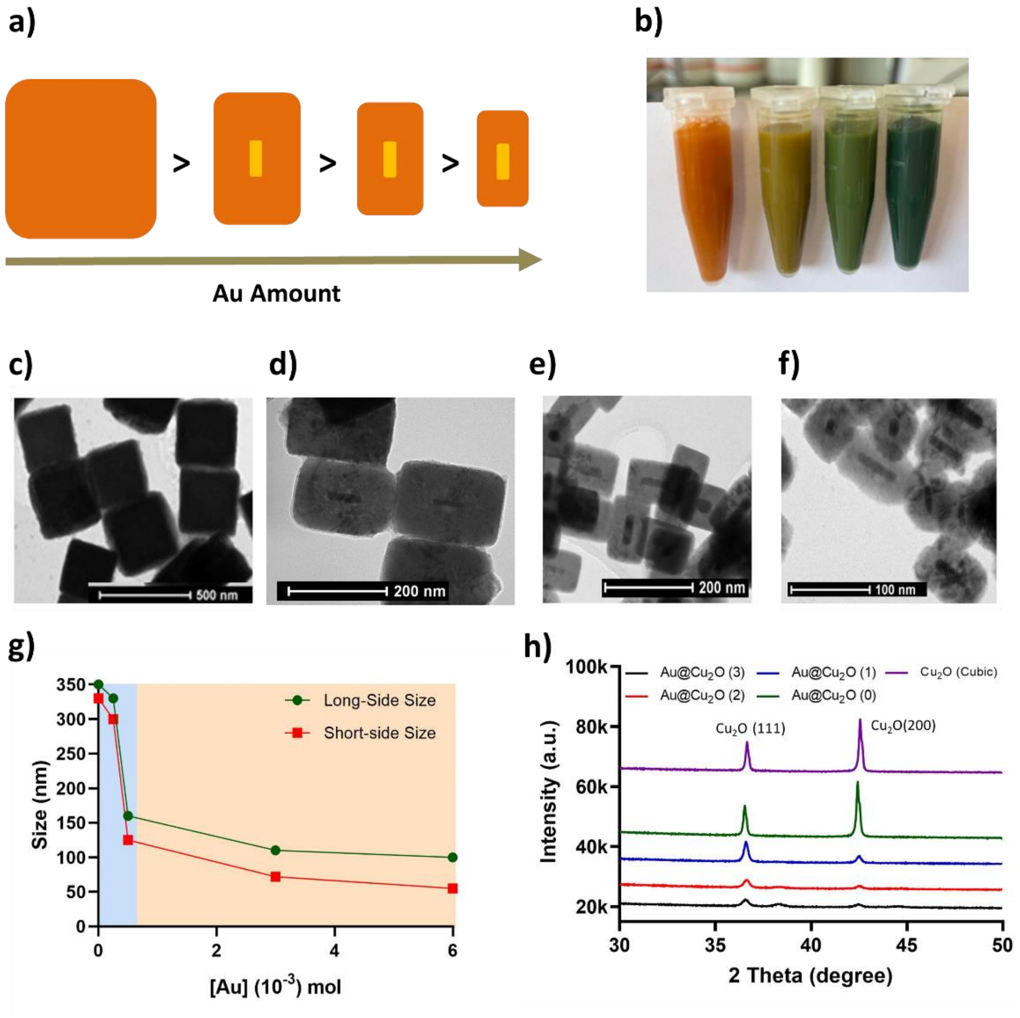


**Figure S1. Characterization of $Cu_2O$-based nanostructures:** a) Schematic illustration of size control of $Cu_2O$-based NPs by using a seed assisted method. b) Image of different $Cu_2O$-based NPs with different color depending size and Au concentration. $Cu_2O$-based NPs are turning green with increasing Au concentrations. TEM characterization of hybrid Au@Cu NPs with core-shell configuration adding c) 0 mmol Au (scale bar = 500 nm), d) 0.5 mmol Au (scale bar = 100 nm), e) 3 mmol Au (scale bar = 200 nm) and f) 6 mmol Au (scale bar = 100 nm). g) Size distribution of Au@Cu NPs with different concentration of Au. h) XRD of Au@Cu NPs with different sizes.

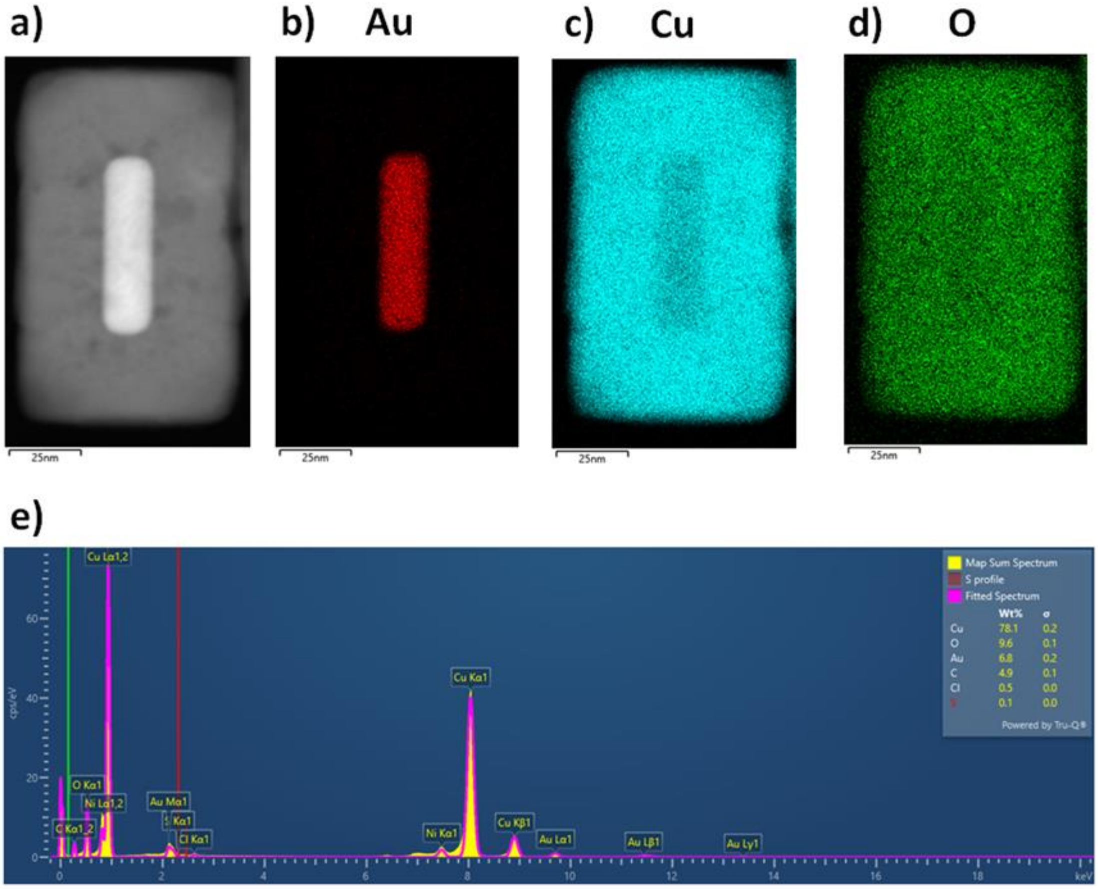


**Figure S2. Additional characterization of core-shell AuNR@$Cu_2O$ nanostructures:** a) HAADF-STEM image and (b-d) elemental analysis mapping by EDX analysis of a single Au@Cu NP where we can observe a cubic shape with a core-shell configuration with the Au nanorod as core and a copper oxide shell. Copper is located around Au core forming a cubic shell (blue) and d) oxygen is co-localized with copper in the cubic shell (green); (scale bar = 25 nm). e) EDX spectra corresponding to a single core-shell Au@Cu composite with an atomic Cu/Au ratio of 35 and a Cu/O ratio of 2.

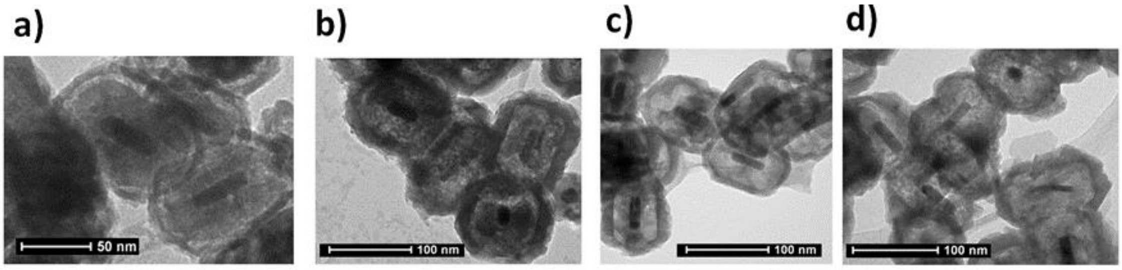


**Figure S3. Sulfidation of Au@$Cu_2$O structures:** Representative TEM images corresponding to different Au@Cu (SS) NPs after treatment with different volumes of $Na_2S$: a) 5 µL; b) 10 µL; c) 20 µL and 50 µL of $Na_2S$ solution (Scale bars = 100 nm).

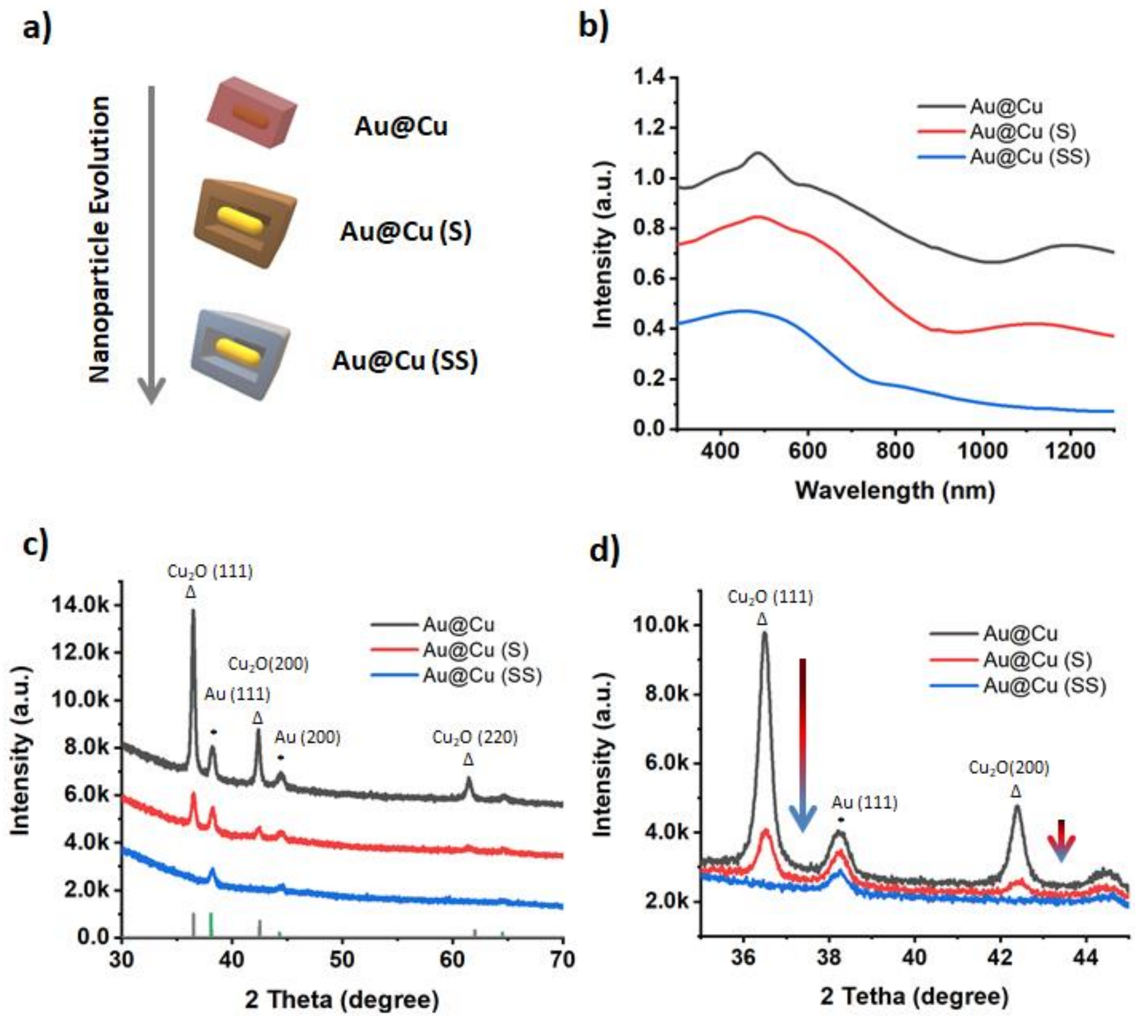


**Figure S4. Characterization of Au@Cu after sulfidation:** a) Schematic illustration of Au@Cu NPs sulfidation under the presence of $Na_2S$ for 30 second and 1h; b) UV-vis spectra evolution of Au@Cu NPs after $Na_2S$ treatment to form Au@Cu (S) and Au@Cu (SS) NPs; c) X-ray diffractograms of Au@Cu, Au@Cu (S) and Au@Cu (SS) NPs accounting for the presence of crystalline Au and $Cu_2O$ phases; d) Detail on the progressive disappearance of (111) and (200) diffraction peaks of the $Cu_2O$ upon sulfidation.

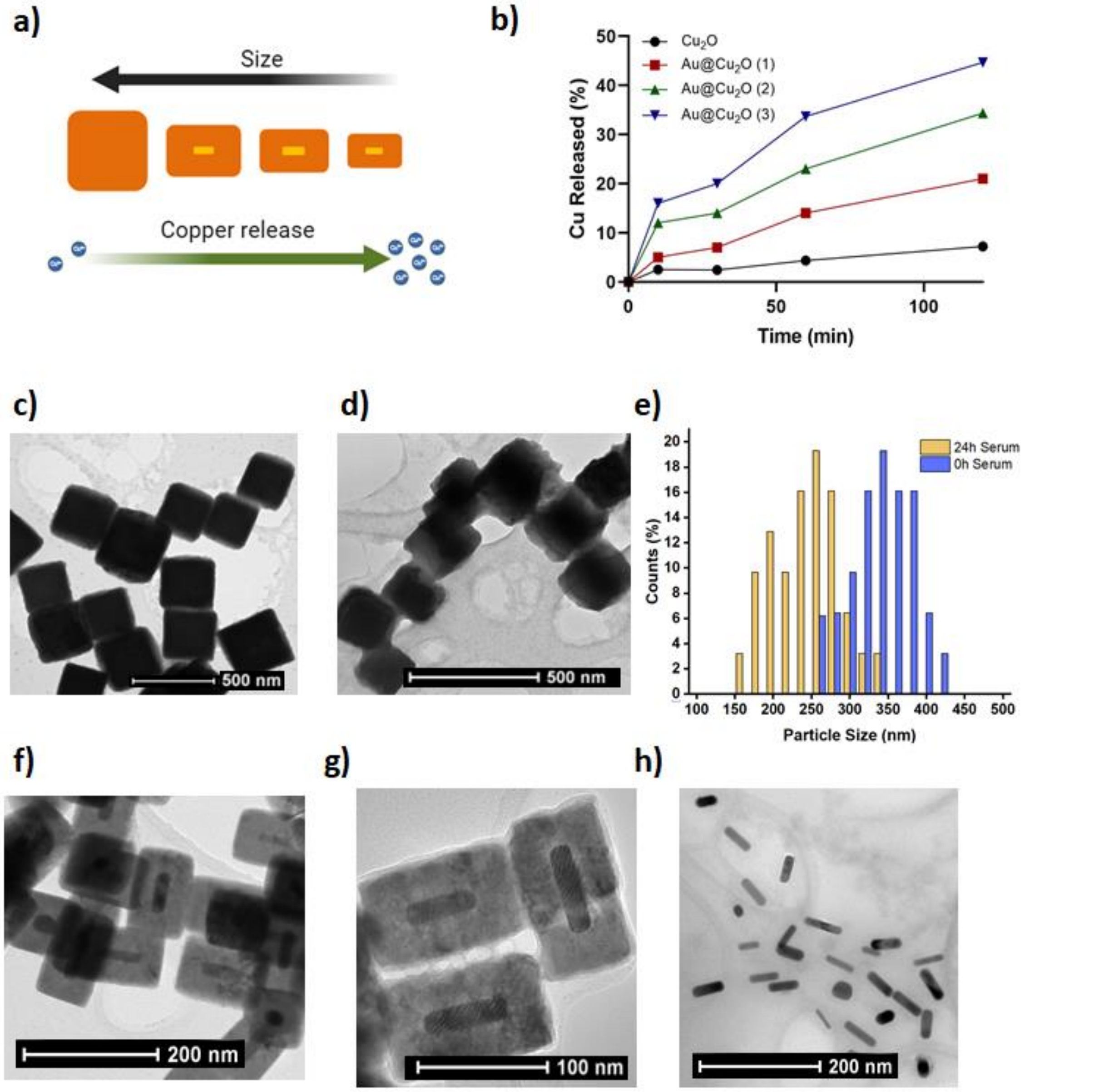


**Figure S5. Copper release kinetics:** a) Schematic illustration of influence of $Cu_2O$-based NPs in copper release kinetics; b) Cu release rate of $Cu_2O$-based NPs with different sizes, c) Representative TEM images of as prepared $Cu_2O$ NPs; d) TEM images of the $Cu_2O$ NPs after exposure to serum for 24 h; e) Particle size distribution before and after serum exposure; f) TEM image of as prepared Au@Cu NPs; g) TEM image of Au@Cu NPs after exposure to serum for 2h and h) 24h, respectively.

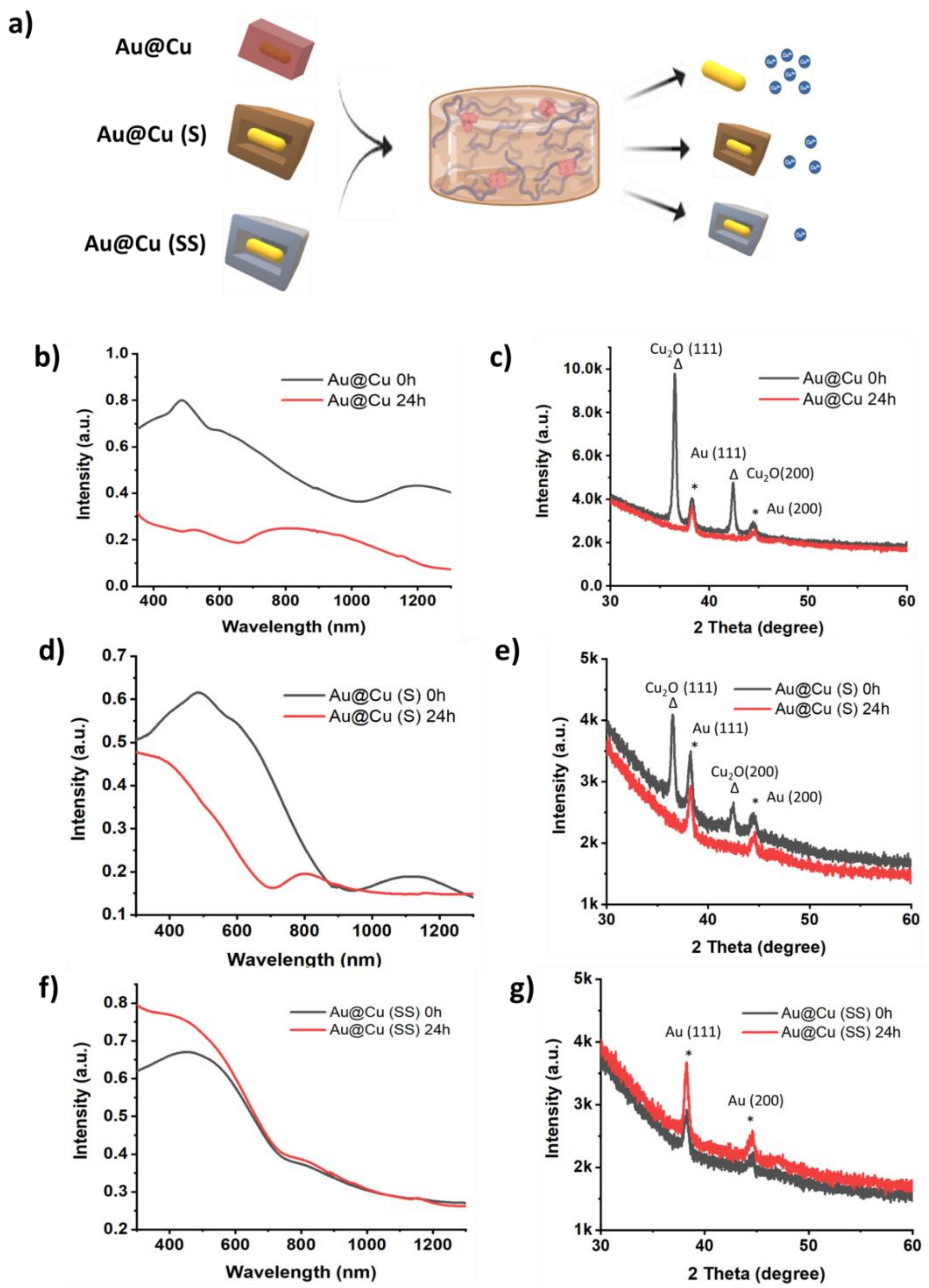


**Figure S6. Characterization of Au@Cu structures before and after exposure to serum:** a) Schematic characterization of Cu release under relevant physiological conditions; b) UV-vis spectra corresponding to the Au@Cu NPs before and after treatment with serum for 24h; c) XRD analysis of the Au@Cu NPs before and after treatment with serum for 24h; d) UV-vis and e) XRD analysis of Au@Cu (S) NPs treated with serum for 24h. f) UV-vis and g) XRD analysis of Au@Cu (SS) NPs treated with serum for 24h.

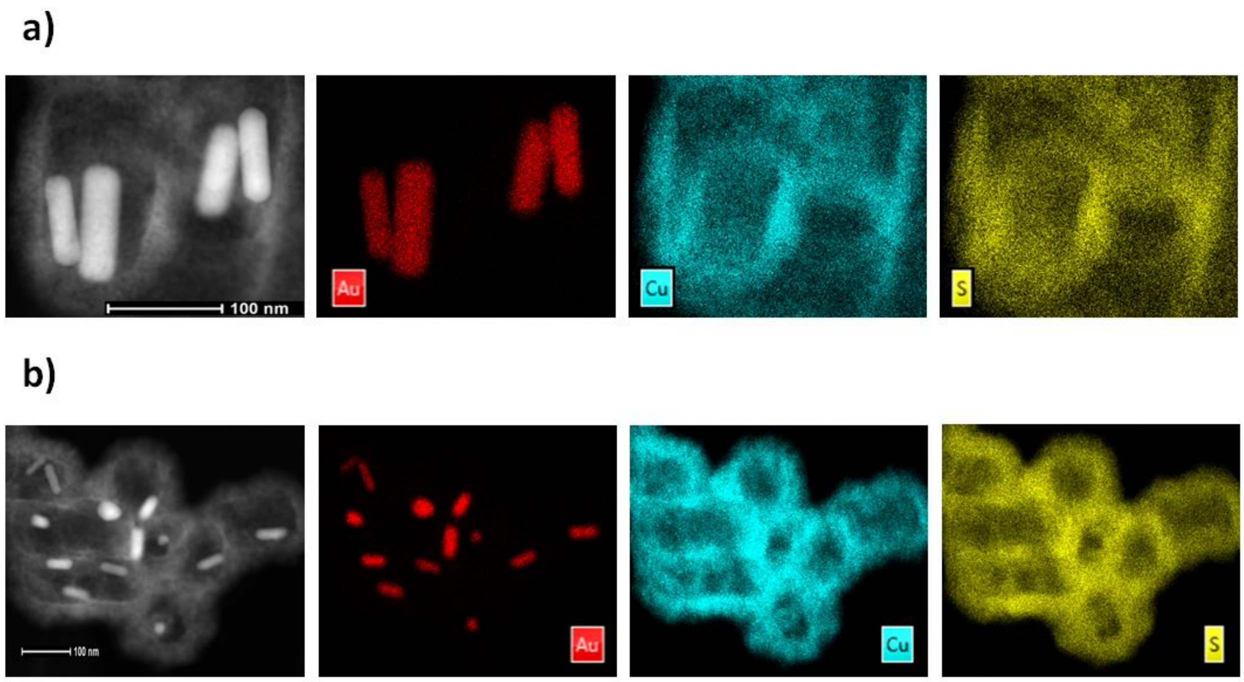


**Figure S7. Additional HAADF-STEM-EDX characterization after serum exposure:** a) Au@Cu (S) NPs after 24h serum treatment with yolk shell configuration where Au is placed in the internal yolk and Cu is removed and placed just co-localized with S in the external shell. b) Au@Cu (SS) NPs after 24h serum treatment with analogous configuration where Cu and S are co-localized in the shell.

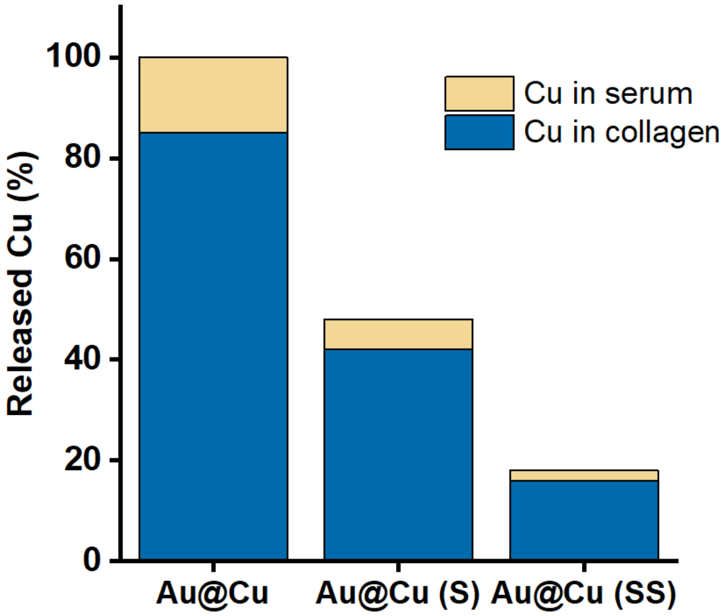


**Figure S8. Study of Cu release in collagen hydrogel:** Effect of hydrogel on Cu ion release from gel phase for Au@Cu, Au@Cu (S) and Au@Cu (SS) NPs in presence of serum after 24h exposure. [Au@Cu-based NPs]: 0.1 mg/mL, collagen density: 6 mg/mL.

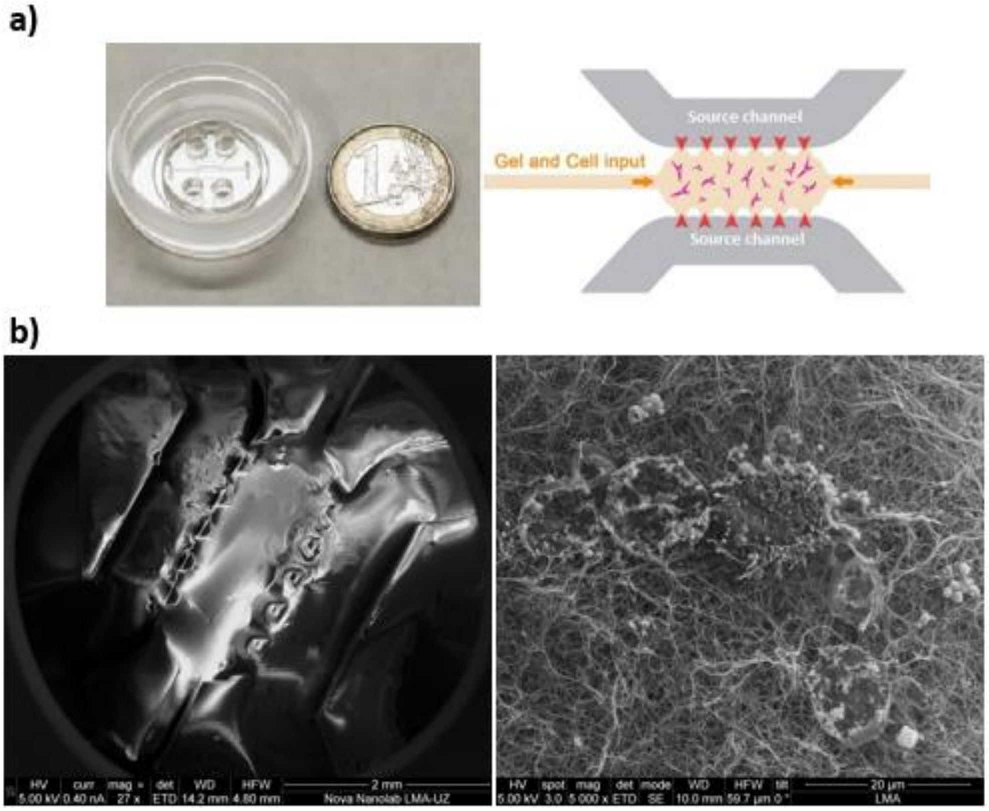


**Figure S9. Configuration of the 3D microreactor devices:** a) Geometry and design of the microfluidic devices described in the present work, with a central chamber containing the gel with the 3D cells embedded and two side channels to feed the nutrients; b) Cryo SEM images of the whole devices (left) and the cells interacting with de filling hydrogel (right).

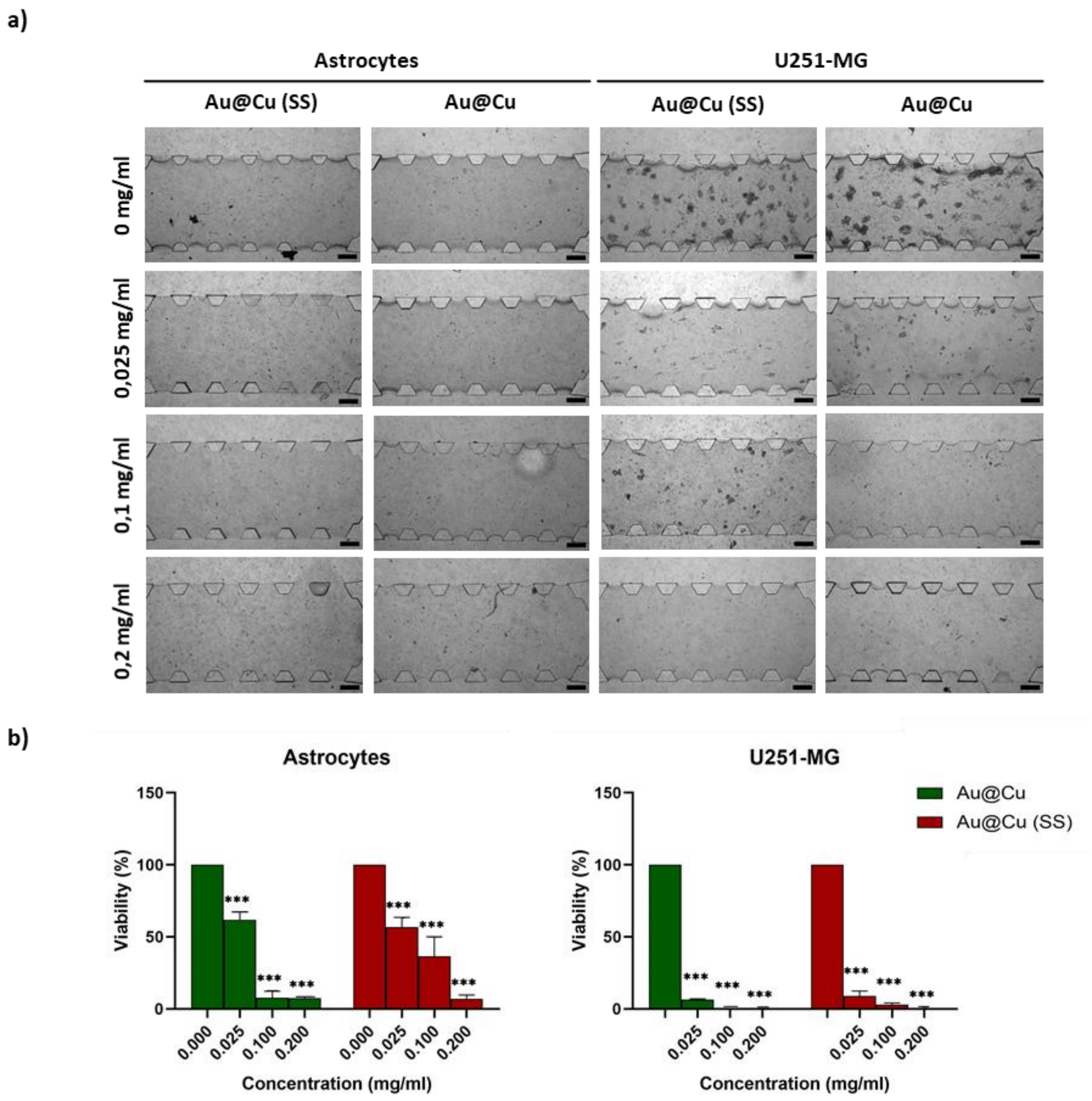


**Figure S10. Cytotoxicity of Au-based NPs in healthy and malignant cells:** a) Brightfield microscopy at day 7 allows the comparison of the growth of the 3D cellular cultures when exposed to a total concentration of 0.2, 0.1, 0.025 mg/mL using Cu-based NPs with different release patterns. Scale bar is 250 µm. (b) CellTiter-Glo® Luminescent Cell Viability Assay. The data were normalized taking into account the control as 100% viability. Data shown as the mean ± SD (n = 3); *p-val < 0.033; **p-val < 0.002; ***p-val < 0.001.

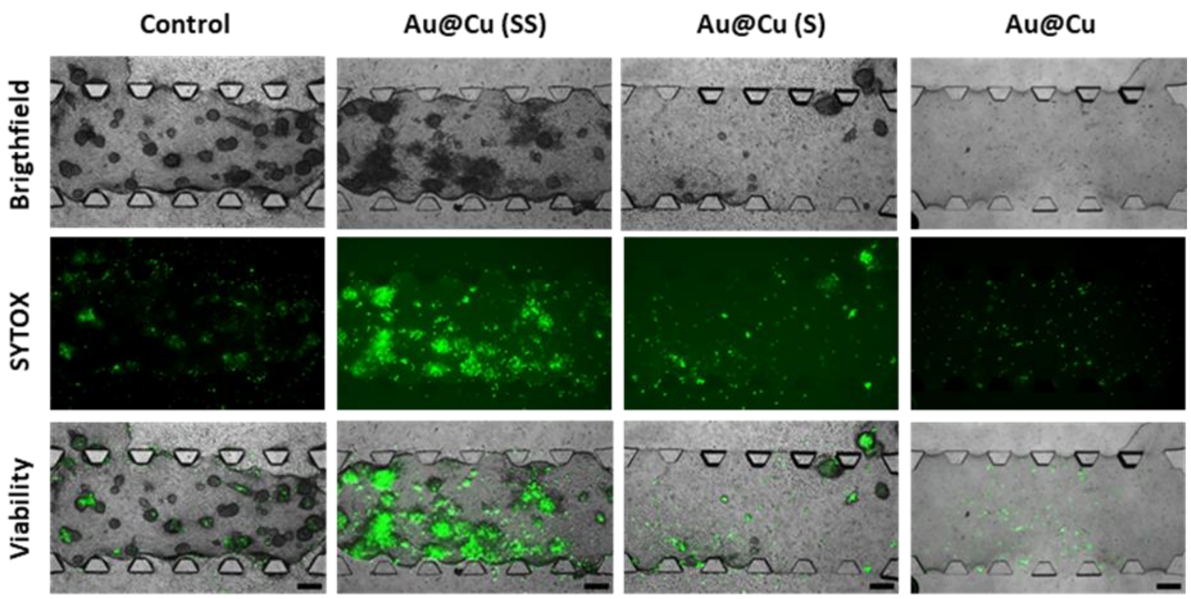


**Figure S11. 2D fluorescence images of viability in GBM spheroids treated with different Cu-based NPs:** Cellular viability tested using SYTOX® Green Nucleic Acid Stain. Scale bar is 250 µm. All fluorescence images were acquired with 488nm (green) laser and have identical exposure times and normalization.

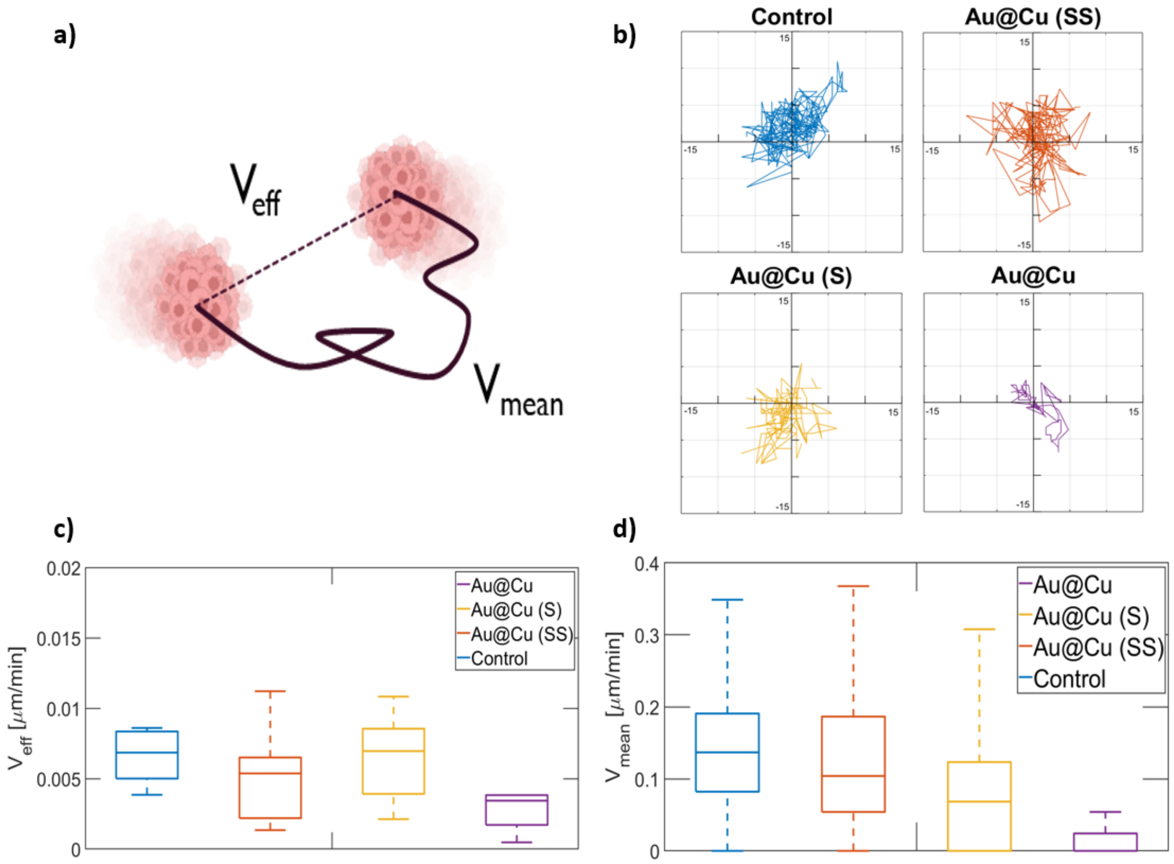


**Figure S12. Progression of the spheroids in the presence of different Cu-based NPs:** a) Schematic representation of mean and effective velocity of spheroids movement; b) Cluster migration data obtained from 12h time lapses; c) Effective (accounting for only the starting and final positions) and d) mean (instantaneous movement) velocities of spheroids treated with different Cu-based NPs (n=6).

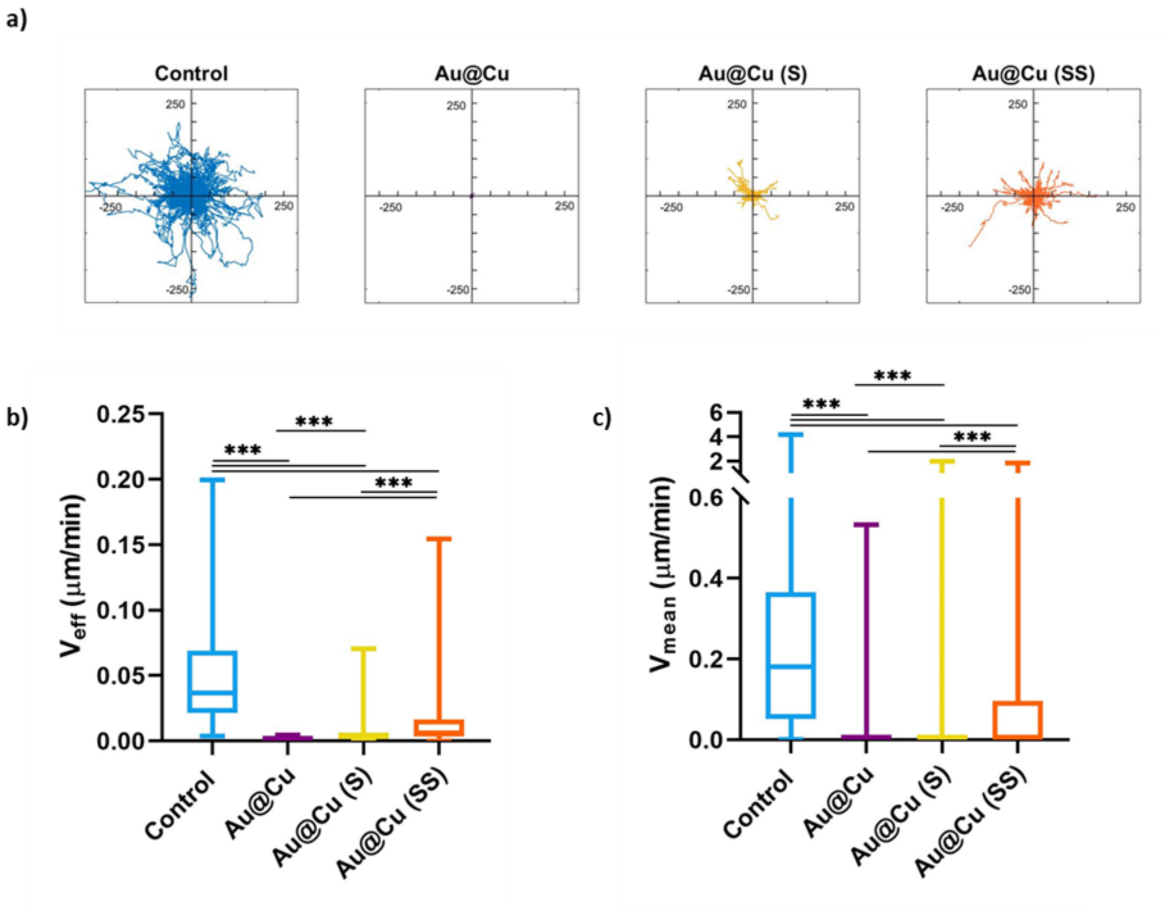


**Figure S13. Cell individual migration in the presence of different Cu-based NPs:** a) Individual cell migration trajectories obtained from 24h time lapses; c) Effective (accounting for only the starting and final positions) and d) mean (instantaneous movement) velocities of cells treated with different Cu-based NPs. Data shown as its distribution with median and the interquartile range (IQR) (n=3; sample size = 50 cells/microdevice); *p-val < 0.033; **p-val < 0.002; ***p-val < 0.001.

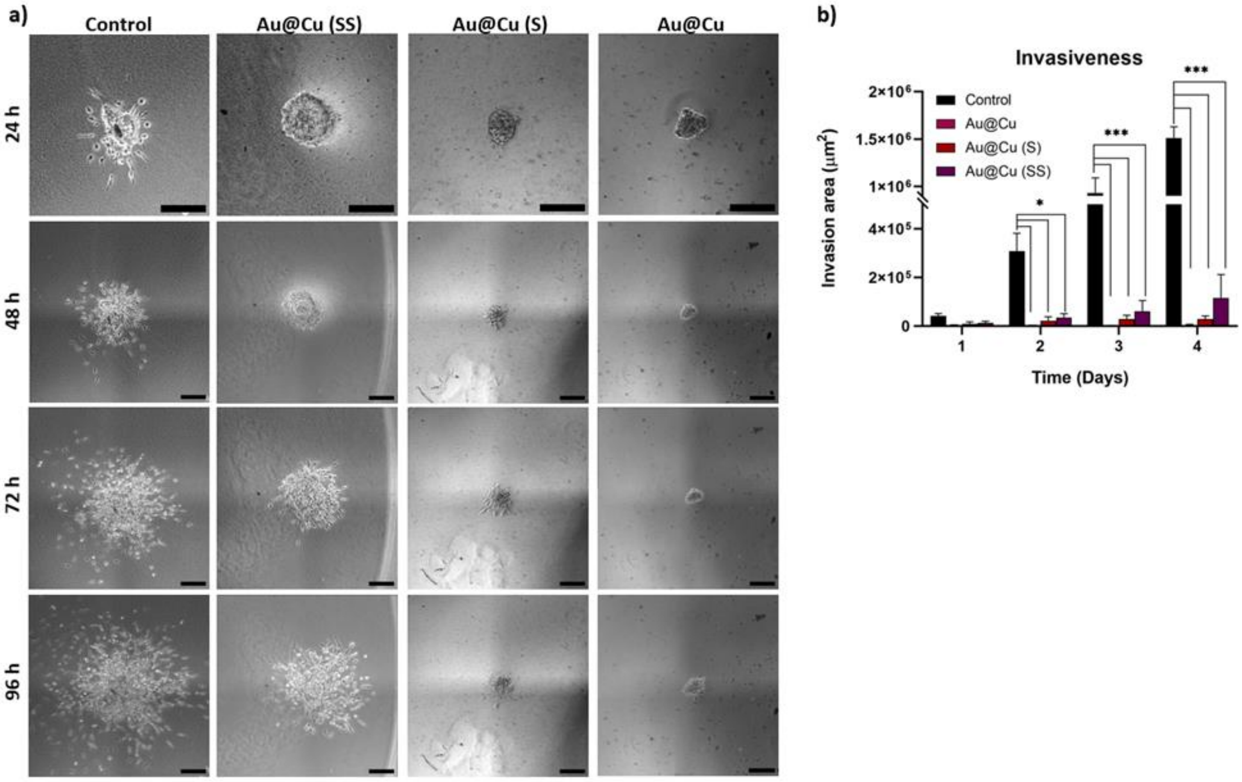


**Figure S14. Cell individual invasiveness in the presence of different Cu-based NPs:** a) Brightfield microscopy along 4 days (n=4) allows the comparison of the cell migration from a spheroid embedded in hydrogel when exposed to a total concentration of 0.1 mg/mL using Cu-based NPs with different release patterns. Scale bar is 200 µm. b) Images where processed and segmented to obtain the invasion area. Data shown as the mean ± sem (n = 4); *p-val < 0.033; **p-val < 0.002; ***p-val < 0.001.

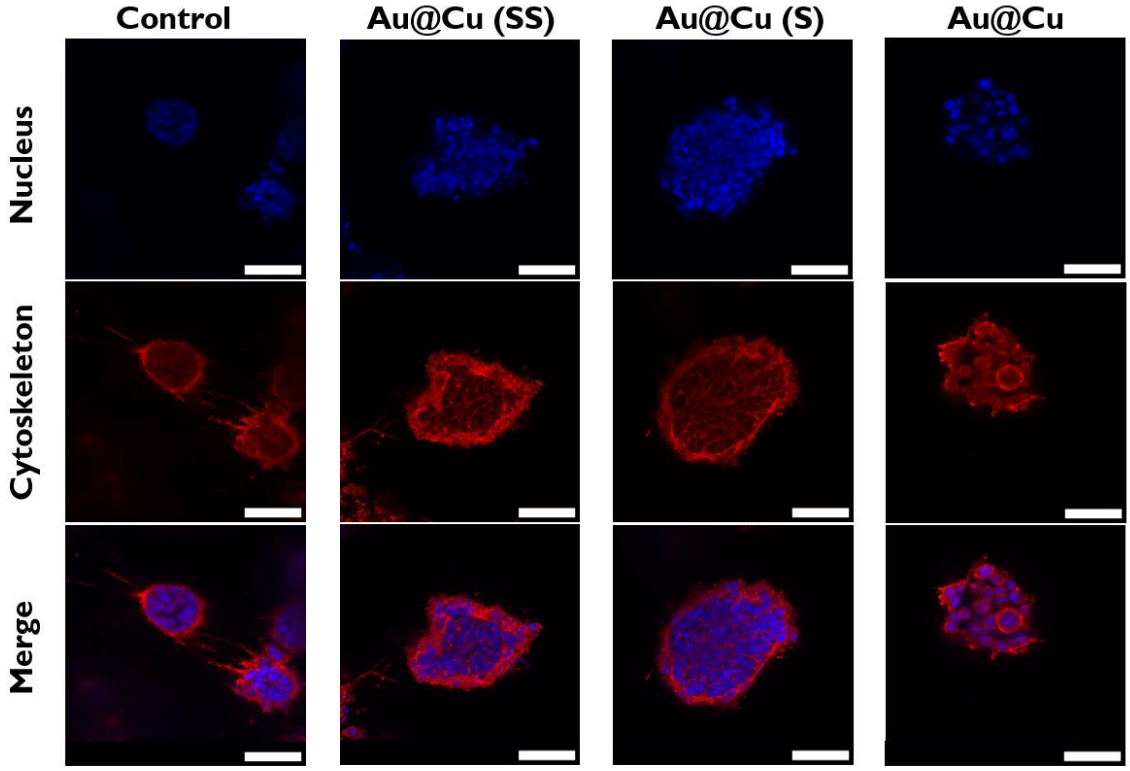


**Figure S15. 2D confocal images of GBM spheroids treated with different Cu-based NPs:** Nuclei was stained with Dapi (blue) and the cytoskeleton was stained with phalloidin (red); Scale bar is 50 µm.

**Movie S1.**

Cluster migration in control condition.

**Movie S2.**

Cluster migration with Au@Cu (SS) NPs treatment.

**Movie S3.**

Cluster migration with Au@Cu (S) NPs treatment.

**Movie S4.**

Cluster migration with Au@Cu NPs treatment.

**Movie S5.**

Cluster migration and death with Au@Cu NPs treatment.

**Movie S6.**

Individual cell migration in control condition.

**Movie S7.**

Individual cell migration with Au@Cu (SS) NPs treatment.

**Movie S8.**

Individual cell migration with Au@Cu (S) NPs treatment

**Movie S9.**

Individual cell migration with Au@Cu NPs treatment

**Movie S10.**

3D reconstruction of control spheroid cytoskeleton.

**Movie S11.**

3D reconstruction of Au@Cu (SS) treated spheroid cytoskeleton.

**Movie S12.**

3D reconstruction of Au@Cu (S) treated spheroid cytoskeleton.

**Movie S13.**

3D reconstruction of Au@Cu NPs treated spheroid cytoskeleton.
